# Operando x-ray absorption spectroscopy unveils light-driven redox dynamics at the semiconductor/cocatalyst interface

**DOI:** 10.1126/sciadv.adx8089

**Published:** 2025-09-19

**Authors:** Raffaello Mazzaro, Alberto Piccioni, Marco Salvi, Pierpaolo Vecchi, Michele Mazzanti, Stefano Caramori, Federico Boscherini, Luca Pasquini

**Affiliations:** ^1^ Department of Physics and Astronomy, Alma Mater Studiorum – Università di Bologna, Viale Berti Pichat 6/2, 40127 Bologna, Italy.; ^2^ Istituto per lo Studio dei Materiali Nanostrutturati (ISMN), Consiglio Nazionale delle Ricerche (CNR), Via P. Gobetti 101, Bologna 40129, Italy.; ^3^ Department of Chemical, Pharmaceutical and Agricultural Sciences, University of Ferrara, Via Fossato di Mortara 17, Ferrara 44121, Italy.

## Abstract

Cobalt-based mixed oxides are widely studied as oxygen evolution reaction (OER) catalysts, yet their role as photoelectrochemical cocatalysts remains debated due to scarce operando studies probing irradiation-induced structural changes. Here, we unveil redox dynamics of cobalt-iron oxide (CoFeO*_x_*) cocatalysts in semiconductor photoanodes for solar water splitting. By combining operando x-ray absorption spectroscopy (XAS) with fixed-energy x-ray absorption voltammetry (FEXRAV) at semiconductor/cocatalyst interfaces, we provide an element-selective probe of Co oxidation states under dark and illuminated conditions. Our results reveal a previously unrecognized interfacial Co state, highlighting interface structure’s role in tuning catalytic activity. We observe light-induced reduction in oxidation state and cathodic shift in Co redox potentials, offering insights into hole transfer and catalytic behavior. Identification of a specific photocatalytic cycle, distinct from dark-state electrocatalysis, advances understanding of how light modulates rate-determining steps in OER. These findings underscore the power of operando x-ray techniques in elucidating interfacial charge transfer and guiding design of more efficient photoelectrochemical systems.

## Teaser

Operando x-rays reveal light-driven changes in CoFeO*_x_* catalysts on semiconductors, uncovering a unique photocatalytic cycle.

## INTRODUCTION

Water oxidation photoanodes based on earth-abundant metal oxide semiconductors are actively studied because of their limited environmental impact, the stability against photocorrosion, and the tunable optoelectronic properties (*1*). BiVO_4_/WO_3_ heterostructures are now emerging as one of the most promising photoanodes, but their activity is limited by surface recombination and sluggish charge transfer kinetics. Cobalt-iron mixed compounds, such as CoFeO*_x_*, proved to effectively enhance photocurrent when coupled to BiVO_4_/WO_3_ photoanodes as water oxidation cocatalysts. The origin of the increased efficiency is still debated, but recent studies suggest an enhancement of charge separation efficiency (*2*), rather than improved water oxidation kinetics, underlying possible charge accumulation on the cocatalyst leading to transient modification of the local structure. Usually, charge transfer dynamics is evaluated through optical and photocurrent spectroscopy analysis, providing deep insight of the transport/transfer mechanism. However, as this is an indirect evaluation of possible charge carriers’ pathways in presence of the specific cocatalysts, operando structural characterization of the cocatalyst is of the utmost importance to achieve a complete mechanistic description of the conversion process. X-ray absorption spectroscopy (XAS) and related techniques are powerful tools for a refined investigation of advanced functional materials and devices. XAS can determine the local atomic and electronic structure (including oxidation state) of ultrathin and highly defective layers, lacking the long-range order required for crystallographic analysis. XAS is also highly compatible to electro- and photoelectrochemical (PEC) operando studies, assuming paramount importance toward the understanding of electrocatalytic processes. Operando methods play a pivotal role in monitoring the authentic operating structure, facilitating a profound mechanistic understanding of the catalytic cycle. As a result, XAS finds wide application for materials used in electro- and PEC water splitting (and related applications), as reviewed by various authors (*3*–*6*). Extensive literature is available on operando XAS characterization of MOOH (M = Ir, Co, Ni, and Fe) upon oxygen evolving reaction (OER) conditions (*7*–*14*) in search of a mechanistic understanding of their role in the catalytic cycle, in particular for the archetypal CoO*_x_*H*_y_*, often referred as “CoPi.” However, the question remains fundamentally unanswered, as the matter is still largely debated, especially with (*15*, *16*) respect to the involvement of Co^IV^ as the active site. Contrasting evidence on the presence of Co^IV^ or Co^III^ are reported in operating electrocatalysts (*17*–*19*), further complicated by the intrinsic structural complexity of thin, low crystallinity samples, typically used to this aim. The debate is also open in mixed Co-Fe oxide electrocatalysts. XAS analysis of CoFeO*_x_* with different Co/Fe ratio (*12*) suggested that Co^IV^ acts as the active site for OER electrocatalysts, while Fe^IV^ participates as a redox cooperative center, enhancing the oxidizing power of the Co site (*20*). On the other hand, Burke *et al.* (*14*) mainly attributed the enhanced OER overpotential in mixed Co_1−*x*_Fe*_x_*(OOH) to the improved conductivity of the CoOOH lattice that acts as a porous, chemically stabilizing host for Fe atoms.

To date, only a few reports on the XAS operando investigation of PEC processes are available, mainly due to the technical challenges to design operando PEC-XAS cells (*21*). Relevant examples include: (i) evidence for specific shallow electronic transitions in hematite photoanodes (*22*); (ii) the structural rearrangement of WO_3_ photoanodes (*23*), compensating for the accumulation of photoinduced electrons in the conduction band (CB) under open circuit conditions; and (iii) photoinduced charge accumulation at the semiconductor electrocatalyst junction in Fe_2_O_3_/NiO*_x_* (*24*, *25*), Fe_2_O_3_/IrO*_x_* (*26*), and Si/IrO*_x_* (*27*) heterostructures. The only report investigating the evolution of Co-based catalysts (*28*) on a semiconductor photoelectrode (hematite) ascertains the presence of Co^IV^ in OER conditions and reports an average oxidation state of about Co^+3.4^ in absence of applied bias, with a relative variation hampered if compared to the one achieved on a FTO substrate for the same catalyst. This is pointing to a possible interaction with the substrate, not further investigated, which may result from nontrivial charge dynamics at the semiconductor/cocatalyst interface.

Here, to achieve structural understanding of the catalyst activity in the actual PEC operating conditions, a custom PEC-XAS cell was specifically designed to allow for operando monitoring of Co-Fe–based cocatalysts deposited on BiVO_4_/WO_3_ photoanodes, upon bias and illumination stimuli that fully replicate the ex situ conditions. The developed setup, providing limited x-ray photons interaction path with the controlled-flow electrolyte (~100 μm), enhances mass transfer and prevents bubbles formation, acting as one of the main experimental limitations to fully replicate PEC conditions in analogue operando setups (*23*). This allows for stable, real-time correlation of the structural features with the PEC response. The activity of CoFeO*_x_* was studied using fixed-potential spectroscopy at the Co K-edge to examine the steady-state local environment and metal oxidation state, and fixed-energy x-ray absorption voltammograms (FEXRAVs) (*29*) to capture element-specific and light-dependent redox dynamics of the metal center. The resulting characterization shows that the local structure of the catalyst in operating conditions differs substantially from the pristine structure and exhibits a dependence on both applied bias and illumination. The structural analysis sheds light on Co-specific states at the interface with the semiconductor and provides a deeper understanding of its beneficial action on the photoexcited charge carrier dynamics studied by intensity-modulated photocurrent spectroscopy (IMPS) and PEC impedance spectroscopy (P-EIS).

## RESULTS

### Photoanodes fabrication

WO_3_/BiVO_4_ photoanodes (hereinafter referred to as BVO) were prepared by a multistep approach (*30*, *31*) and coated with increasing thickness of CoFeO*_x_* as cocatalyst, as discussed in Materials and Methods. Figure S1 shows the porous morphology of BVO characterized by interconnected nanoparticles with typical diameter in the 50 to 100 nm range. Previous high-resolution observations revealed that BiVO_4_ is constituted by ultrafine nanocrystallites supported on WO_3_ (*32*). Two deposition times for CoFeO*_x_* were selected, to assess the influence of the cocatalyst thickness. The photoanodes are thus referred to as 1 nm@BVO and 10 nm@BVO, according to the estimated thickness of the CoFeO*_x_* layer (fig. S2). It should be noted that the reported thickness values are indicative, especially in the case of the 1-nm sample, where the intended goal was to achieve a submonolayer- or monolayer-equivalent deposition, an extremely challenging task to characterize with conventional techniques. A thick reference CoFeO*_x_* layer was also deposited on FTO (10 nm@FTO), to assess the role of the substrate on cocatalyst function.

### PEC characterization

**Fig. 1.**
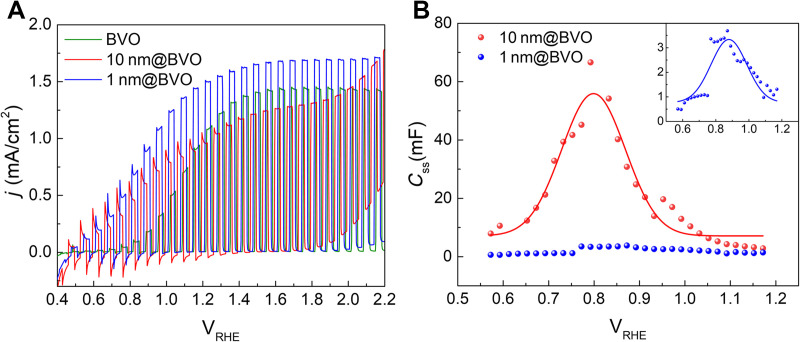
PEC characterization of photoanodes. (**A**) Chopped linear sweep voltammetries under 1 sun illumination of a BVO photoanode superimposed to those of 1 nm@BVO and 10 nm@BVO. Scan rate 50 mV/s. (**B**) Surface state capacitance associated with the CoFeO*_x_* obtained by P-EIS. The inset reports an enlarged view of the thin overlayer case.

The PEC measurements were performed in borate buffer 0.1 M solution at pH 9 with Na_2_SO_4_ 0.5 M as supporting electrolyte; the potential scale in volts versus RHE (V_RHE_) is used throughout. Figure 1A reports the linear sweep voltammetry under chopped AM 1.5G illumination of a bare BVO photoanode at pH = 9 (green line), superimposed to the ones measured for 1 nm@BVO and 10 nm@BVO.

The PEC performance is remarkably improved by the addition of a thin CoFeO*_x_* layer (1 nm@BVO), as shown by a ≈ 400 mV cathodic shift of the onset potential and a ≈ 20% increase of the saturation photocurrent. The deposition of a thicker cocatalyst (10 nm@BVO) results in the same onset potential shift, but the saturation photocurrent decreases compared to the bare photoanode. In the +0.5 to 1 V_RHE_ region, the photocurrent transients show cathodic decays consistent with slow recombination involving surface states. The recombination of holes trapped in these surface states with photoexcited electrons in the CB or shallowly trapped electrons is avoided when the voltage increases above ca 1 V, at which the photocurrent approaches the plateau value. Notably, the recombination transients are largest for 10 nm@BVO: This suggests that hole transfer to the electrolyte becomes difficult in the presence of a thick cocatalyst overlayer, leading to enhanced back recombination with photoexcited electrons. The cocatalyst overlayers also lead to an anticipated onset of the dark current, which starts at about +1.8 V_RHE_ and is clearly enhanced by increasing the amount of CoFeO*_x_*, consistent with its catalytic effect on the OER. The cyclic voltammetries (CVs) measured in the dark on the same samples show a broad cathodic wave in the <1.0 V_RHE_ region (fig. S3). This is the result of two processes: the reduction of V^V^ to V^IV^ within the BiVO_4_ layer and the W^VI/V^ reduction within WO_3_.

The incident photon–to–current conversion efficiency (IPCE) shown in fig. S4A shows that the highest quantum yield (ca. 40%) is achieved with the 1 nm@BVO photoanode, in agreement with the *j*/*V* characteristics. The action spectra of all photoanodes exhibit the same spectral response range starting at ca. 500 nm, ruling out any direct photoactivity of CoFeO*_x_* in creating photoinduced charge separation. This result confirms that CoFeO*_x_* has to be regarded as an electrochemically active but photochemically inactive overlayer. The onset of photoactivity in the IPCE spectra coincides with the one of optical absorbance as shown by the ultraviolet-visible (UV-vis) spectra in fig. S4B.

P-EIS was performed with the aim of investigating the interfacial capacitance of the photoanodes. The experimental procedure and the equivalent circuit models used to fit the data are detailed in the Supplementary Materials. The analysis of P-EIS data show that the capacitance associated with the cocatalyst has a bell-shaped distribution, the height of which correlates positively with the cocatalyst thickness (Fig. 1B). The shape is typical of a chemical capacitance $C_{\text{SS}}$ arising with a distribution of surface states. $C_{\text{SS}}$ is peaked around 0.8 to 0.9 V and spans the 0.6 to 1.0 V interval, i.e., the region where recombination decays characterize the photocurrent transients (Fig. 1A).

**Fig. 2.**
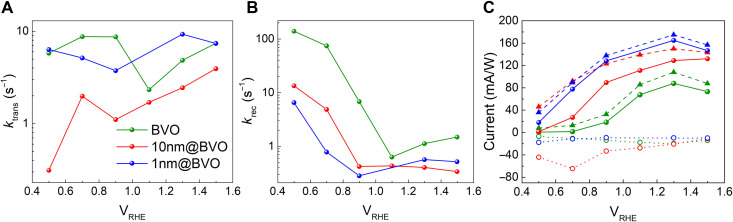
IMPS analysis. (**A**) Transfer rate, (**B**) recombination rate, and (**C**) Gartner current (dashed line), recombination current (dotted line), and total photocurrent (solid line) determined by analysis of IMPS data for the three photoanodes. Legend is showed only for (A) but colors are the same across the three pictures.

IMPS was carried out to disentangle the charge separation, transfer, and recombination kinetics that determine the PEC properties of the photoanodes. The IMPS data in fig. S8 were fitted with a recently developed software that extracts the distribution of relaxation times using a Lasso regression algorithm (*33*) and calculates the rate constants $k_{\text{trans}}$ and $k_{\text{rec}}$. Figure 2A shows that a thin cocatalyst overlayer does not modify the transfer rate, as $k_{\text{trans}}$ of 1 nm@BVO is similar to the one of bare BVO. However, $k_{\text{trans}}$ decreases substantially in 10 nm@BVO, indicating poor transport properties of the thick CoFeO*_x_* layer. On the other hand, Fig. 2 (B and C) shows that, independently on its thickness, the cocatalyst decreases the recombination rate $k_{\text{rec}}$ and increases the Gartner’s current, which describes charge separation within the photoanode on fast timescales. The large recombination current observed in 10 nm@BVO originates with the increased ratio $k_{\text{rec}}/k_{\text{trans}}$ and indicates that a higher fraction of holes recombines before being transferred to the electrolyte. The combination of P-EIS and IMPS therefore suggests that the principal action of the cocatalyst consists in suppressing electron-hole recombination both on a fast timescale (< 1μ*s*) within the semiconductor and on a slower timescale at the semiconductor/electrolyte interface, rather than in boosting hole transfer to the electrolyte. This picture agrees with previous IMPS studies on Co-Pi catalyzed BVO (*34*) and transient absorption spectroscopy of photoanodes similar to 1 nm@BVO (*31*). It has been proposed that the deposition of Co-based overlayers passivates BVO surface states leading to Fermi level unpinning, which enhances band bending and facilitates charge separation. In the next sections, thanks to the unique element-selective capability of operando XAS, we will provide atomistic evidence of photoexcited hole transfer toward Co centers.

### Structure of the cocatalyst: Effect of electrolyte exposure

Local structural characterization of the cocatalyst was performed by XAS at the Co K-edge using a custom-developed flow PEC cell with ultrathin kapton window for operando experiments, as detailed in the Supplementary Materials. X-ray absorption near-edge structure (XANES) and extended x-ray absorption fine structure (EXAFS) regions of the spectrum were registered for the samples in different environmental conditions to probe the oxidation state of Co and the local coordination, respectively. EXAFS data were fitted using CoOOH-heterogeneite as reference phase, based on previous studies on similar compounds (*13*). The details of the analysis and the fitting results for the first two coordination shells (Co-O and Co-Co) are reported in Materials and Methods and the Supplementary Materials, respectively (fig. S10 and table S1).

**Fig. 3.**
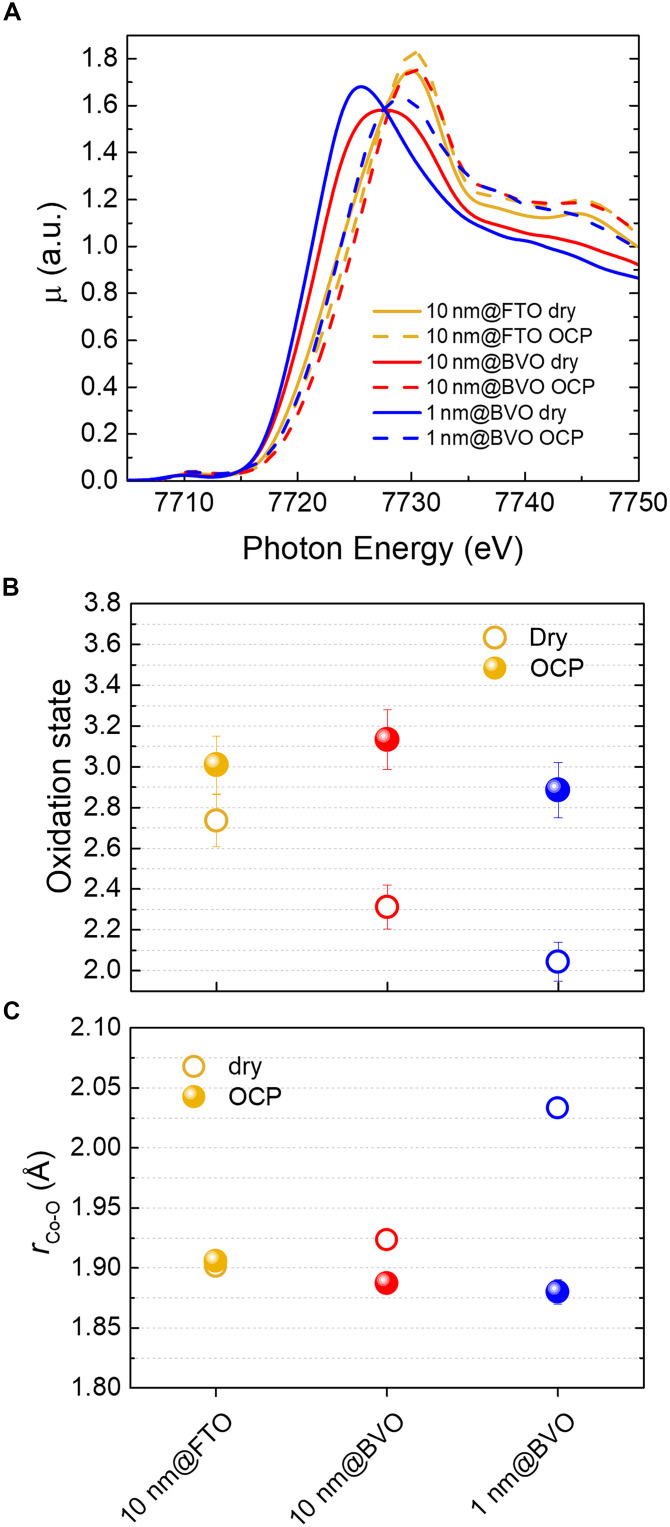
In situ XAS analysis upon exposure to the electrolyte. (**A**) Normalized Co K-edge XANES of pristine 1 nm@BVO, 10 nm@BVO, and 10 nm@FTO in dry conditions (dry), and submerged in the electrolyte (pH 9) at OCP (1.1 V_RHE_). (**B**) Co oxidation state calculated from the absorption edge for the reported samples. (**C**) Co─O interatomic distance from fitting the first Co─O coordination shell visible in FT-EXAFS. The error bars for the coordination numbers and Co─O distances are provided by the standard EXAFS fitting procedure, while the ones for Co oxidation state were calculated from the energy resolution of 0.1 eV and the integration of the absorption edge.

Figure 3A reports the comparison between the Co K-edge XANES for 1 nm@BVO, 10 nm@BVO, and 10 nm@FTO in the dry state, and submerged in the electrolyte within the flow PEC cell in the dark at open circuit potential (OCP), which is close to +1.1 V_RHE_. Figure 3B displays the corresponding Co oxidation state determined from the absorption edge energy. To this purpose, a calibration curve was obtained by measuring reference compounds, which resulted in a ~2.5 eV shift per oxidation state unit in full agreement with previous literature (*17*, *35*). The absorption edge energy was calculated by means of the integral method (*36*). This procedure yields an accurate relative comparison between different measurements, while the absolute value of the oxidation state is affected by a typical uncertainty of about ±5%, as represented by the error bars in the figure, due to some degree of arbitrariness in the choice of the integration region.

The XANES of dry 10 nm@FTO almost perfectly matches the one reported for CoFeO*_x_* (*11*) at negligible Fe % content, suggesting that the presence of Fe has little or no effect on the local density of electronic states around Co. In comparison, the XANES of dry 1 nm@BVO and 10 nm@BVO exhibits a significant shift of the K edge, which is reflected in a lower oxidation state (Fig. 2B) equal to Co^+2.0^ and Co^+2.3^, respectively. The thickness-dependent oxidation state is suggestive of the influence of the local interfacial environment on the electronic properties of the cobalt centers. In particular, we expect the first layers of cobalt to be chemically attached to oxygen atoms bridged to V^V^ centers forming bonds of the type [Co^X^-O-V^V^] where X is the formal oxidation state of cobalt which varies between II and III in our case. The resulting change in the potential for the associated redox transition is explained by the inductive effect (*37*) of the metal cation in this newly formed species, which is directly correlated to its electronegativity in the corresponding oxidation state (*38*). The strong electron-accepting character of V^V^ decreases the electron donation capability of the bridging O atoms, resulting in the stabilization of the lowest oxidation state of Co (i.e., Co^II^ in our case). In thicker layers these interfacial effects are mitigated, since a larger population of Co centers is remotely attached to the semiconductor surface via structural motifs of the type Co^X^-O-Co^Y^ or Co^X^-O-Fe^Y^ where X and Y can be either II or III, which occur in the bulk of the thicker film. Both Co^II/III^ and Fe^II/III^ are weaker electron acceptors than V^V^ making the bridging oxygen atoms stronger electron donors for the stabilization of Co^X^ in its highest common oxidation state (Co^III^).

Upon exposure to the electrolyte at OCP, the difference between XANES (Fig. 3A) becomes less evident and the oxidation state in all photoanodes stabilizes to approximately Co^+3.0^. The spontaneous restructuring at the CoFeO*_x_*/electrolyte interface thus appears to counteract the substrate-dependent Co oxidation state observed in pristine photoanodes in the dry state. The OCP potential lies in a narrow potential region ≈ + 1.1 V_RHE_ for all photoanodes, resulting from the equilibration of the Fermi level of both cocatalyst and semiconductor with the redox species in the electrolyte. Therefore, the exposure to the electrolyte induces a larger increase of the oxidation state on the overlayers deposited on BVO compared to the one on FTO. This is likely due to the coordination of OH^−^ to the labile sites of the cobalt centers. The oxygen in OH^−^ is a stronger electron donor than the bridging oxygen groups, since the former has a higher electron density, therefore it stabilizes Co^III^. Furthermore, this effect is expected to be more notable in defective thinner layers, which offer a higher density of labile coordination sites where OH^−^ can bind. These labile sites, which are either uncoordinated or coordinated by water molecules, are certainly less represented in thicker layers where most Co centers are surrounded by bridging O groups in an octahedral environment.

The Co-O interatomic distance $r_{\text{Co}-O}$ determined from EXAFS analysis (Fig. 3C) is fully consistent with the oxidation state change, showing a strong shrinking ( 2.03→1.88Å ) in 1 nm@BVO, a milder one ( 1.92→1.88Å ) in 10 nm@BVO, and almost no change in 10 nm@FTO ( ≈1.90Å ). The value at OCP for 10 nm@FTO agrees with $r_{\text{Co}-O}$ = 1.902 Å measured in CoOOH (*13*), while for both CoFeO*_x_* overlayers deposited on BVO it is slightly lower. Since all samples exhibit approximately the same Co oxidation state at OCP, this result suggests a slight distortion of the local structure due to interaction with BVO.

### Potential-induced variations of Co oxidation state

**Fig. 4.**
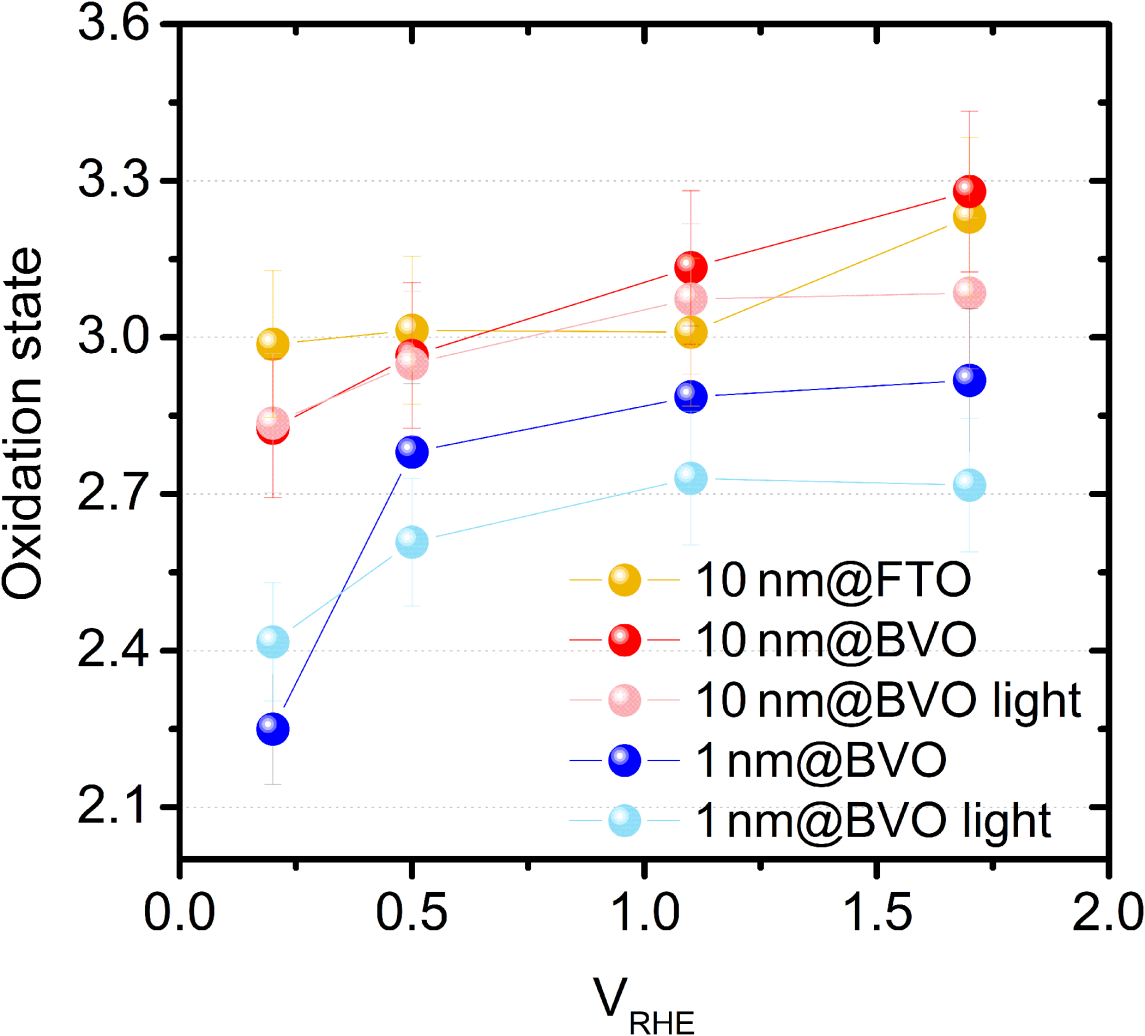
Operando PEC-XAS analysis. Co oxidation state calculated from the absorption edge as a function of the applied potential in light and in dark. Error bars were calculated from the energy resolution (0.1 eV) and the integration of the absorption edge.

Figure 4 reports the oxidation state determined from XANES spectra recorded at different potentials (fig. S11), both in the dark and under AM 1.5G irradiation, in steady-state conditions after a stabilization period of about 5 min. The data points at +1.1 V in the dark actually correspond to those already reported in Fig. 3 at OCP. We notice that the thick cocatalyst on FTO and the thin cocatalyst on the semiconductor exhibit a markedly different behavior in the dark. In 10 nm@FTO, the oxidation state changes in the anodic region above +1.1 V_RHE_, while it remains constant at lower potentials. On the contrary, in 1 nm@BVO the oxidation state stays almost constant above +1.1 V_RHE_ but decreases substantially in the cathodic region, in particular below +0.5 V_HRE_. In the thick cocatalyst on semiconductor (10 nm@BVO), the oxidation state changes over the entire potential range, suggesting a mixed behavior. All these variations correspond to blue shifts (for oxidation) and red shifts (for reduction) of the absorption edge, as clearly visible in fig. S11 (inset).

**Fig. 5.**
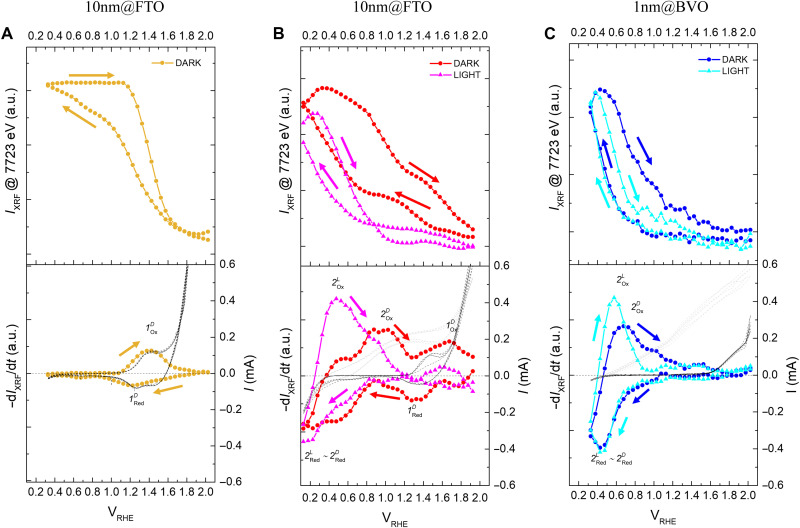
FEXRAV analysis and comparison with conventional voltammetry. FEXRAV experiments on (**A**) 10 nm@FTO, (**B**) 10 nm@BVO, and (**C**) 1 nm@BVO. The graphs report the Co-Kα fluorescence intensity $I_{\text{XRF}}$ (top), $-dI_{\text{XRF}}\;/\;\mathrm{dt}\;$(bottom, left axis), and the measured electrochemical current (bottom, right axis). For the latter the signal in dark is the darker line, while the one under illumination the light-colored line.

This chemical shift of the absorption edge can also be exploited to further investigate the change in oxidation state with applied potential by means of FEXRAV (*29*). FEXRAV consists in fixing the incident photon energy at the point of maximum derivative of the XANES spectrum recorded at OCP and then measuring the x-ray fluorescence intensity ($I_{\text{XRF}}$) as a function of the applied potential. The data have been obtained from the average of several scans, both in the dark and under illumination, as detailed in the Supplementary Materials (fig. S13), while measuring the electrochemical current at the same time. It is expected that $I_{\text{XRF}}$ decreases when scanning in anodic direction due to the blue shift of the absorption edge coupled to Co oxidation, with the reverse occurring in cathodic scans due to Co reduction. The top panels of Fig. 5 confirm this expectation and reveal more clearly the regions where Co-specific redox processes occur.

Furthermore, since $I_{\text{XRF}}$ changes linearly with the integrated amount of negative charge that produces the electrochemical red/ox of the metal center, its negative time derivative $-dI_{\text{XRF}}/\mathrm{dt}$ can be compared to the current measured in standard voltammetry. This data analysis method, previously proposed for spectroelectrochemical characterization (*11*), yields a positive peak corresponding to the maximum oxidation rate during anodic scans and a negative peak for the maximum reduction rate in cathodic scans, resulting in an element-selective voltammetry. The bottom panels of Fig. 5 report the comparison between $-dI_{\text{XRF}}/\mathrm{dt}$ and the electrochemical current as a function of the applied potential. As a result, FEXRAV analysis enables precise correlation between the (photo)electrochemical activity and the redox behavior of the cocatalyst, by providing element-selective, potential-resolved monitoring of redox processes, capabilities that cannot be fully achieved through XANES measurements at discrete potentials.

In sample 10 nm@FTO (Fig. 5A), most of the $I_{\text{XRF}}$ variation takes place between 1.1 V_RHE_ and 1.7 V, consistent with the range where the transition from Co^+3.0^ to Co^+3.2^ occurs in Fig. 4. Consequently, the FEXRAV derivative $-\frac{dI_{\text{XRF}}}{\mathrm{dt}}$ shows an anodic wave labeled as $1_{\text{Ox}}^{D}$ at ~ +1.40 V_RHE_ and a cathodic wave labeled as $1_{\text{Red}}^{D}$ at ~ +1.25 V, which yield an equilibrium potential $E_{\text{eq}}$ = +1.33 V. The FEXRAV waves match quite well those observed in CVs (bottom of Fig. 5A) and can be assigned to the Co^II^/Co^III^ redox reaction. A similar behavior was previously observed for the CoCat structure (*17*), where a potential increase from +1.3 to +1.8 V_RHE_ corresponds to an estimated change from Co^+2.6^ to Co^+3.2^.

Differently, in sample 10 nm@BVO, $I_{\text{XRF}}$ changes over the entire potential window without reaching a plateau (Fig. 5B). The derivative (bottom panel) shows two main processes at different potentials, labelled as $1_{\text{Ox}}^{D}$/$1_{\text{Red}}^{D}$ and $2_{\text{Ox}}^{D}$/$2_{\text{Red}}^{D}$ . The former has a counterpart in the voltammogram just before the rise of the dark current, resembling the behavior of 10 nm@FTO. Therefore, $1_{\text{Ox}}^{D}$/$1_{\text{Red}}^{D}$ can be assigned to the Co^II^/Co^III^ oxidation, although its equilibrium potential $E_{\text{eq}}=+1.5$ V_RHE_ is slightly shifted toward more anodic values. The $r_{\text{Co}-O}$ interatomic distances (fig. S12 and table S1) are also a function of the applied potential, in full agreement with the interpretation of the XANES spectra and FEXRAV analysis. A compression (expansion) of $r_{\text{Co}-O}$ and a decrease (increase) of the N_Co-O_ coordination number is observed, concurrently with oxidation (reduction) at potentials surrounding the $1_{\text{Ox}}^{D}$/$1_{\text{Red}}^{D}$ process.

The second FEXRAV process ( $2_{\text{Ox}}^{D}$/$2_{\text{Red}}^{D}$ ) clearly indicates that part of the Co centers on BVO undergoes a second redox reaction in a potential range where it would be chemically stable on a conductive substrate, like FTO. Consistently, following $2_{\text{Red}}^{D}$ , the oxidation state decreases as displayed by XANES analysis reported in Fig. 4. Notably, the process does not have a clear counterpart in the voltammogram, although a broad reductive wave is visible in the interval from +1.0 to +0.4 V_RHE_ (Fig. 5B and fig. S3). As discussed above, this wave arises from the filling of V-related intragap states and anticipates the reduction of W^VI^ to W^V^. (*23*)

The disparity between the reversibility of the $I_{\text{XRF}}$ signal and the voltammogram’s behavior can be attributed to the kinetics of charge trapping and release. These processes are mediated by the surface states of BiVO_4_ under the given potential regime. During the cathodic sweep, the charge trapping mechanism involves the reduction of V^V^ to V^IV^ within the BiVO_4_ lattice, concomitant with the formation of surface oxygen vacancies (*39*). The occurrence of the FEXRAV $2_{\text{Ox}}^{D}$/$2_{\text{Red}}^{D}$ wave highlights a rapid charge exchange with the Co-based cocatalyst within the same potential region. Analysis of the $r_{\text{Co}-O}$ interatomic distance (fig. S12 and table S1) indicates no significant expansion during the reduction process. This suggests the formation of a strained structural configuration, potentially due to the release of a neighboring oxygen atom from the interfacial bridging V─O─Co oxo bond, as corroborated by a reduced coordination number. This structural adjustment provides a plausible explanation for the absence of a similar phenomenon on FTO-supported CoFeO*_x_* under equivalent potential conditions. Upon reversal of the potential sweep toward the anodic direction, no anodic current is detected, irrespective of the presence of the cocatalyst. This observation points to a slow charge detrapping process (*40*), where charges are released from BiVO_4_ surface states to the conduction band. Such sluggish detrapping dynamics underpin the surface recombination–limited activity characteristic of BiVO_4_ photoanodes (*34*). Conversely, the process observed in the FEXRAV analysis is completely reversible within the same potential region, underscoring the capability of the cocatalyst to facilitate rapid charge exchange with defective surface states in the underlying semiconductor. This indicates that Co centers enhance the spatial separation of electrons trapped in BiVO_4_ surface states by efficiently scavenging the charges stored in the BiVO_4_ layer. This conclusion is further supported by the observed increase in *C*_ss_ as a function of cocatalyst thickness (Fig. 1B), which can be attributed to greater coverage of surface states with increasing thickness. The concurrent improvement in the Gartner current and reduction in the recombination rate within the same potential region are also consistent with this interpretation.

It is important to note, however, that the heterogeneity of the interfacial environment on BiVO_4_ likely results in a distribution of redox states due to locally varying coordination conditions, in particular in the thicker samples. Given that the charge trapping process is expected to be interface-limited, it can be inferred that Co sites not in direct contact with BiVO_4_, specifically those exhibiting the conventional Co^II/III^ redox behavior, do not actively participate in the trapping process. Instead, these sites likely act as a barrier for surface charge extraction, thereby reducing film conductivity, consistent with the observed decrease in charge transfer rates for thicker CoFeO*_x_* layers.

This suggests that the spatial separation of charges, while enhanced by the active Co sites in direct contact with BiVO_4_, is hindered by the presence of outer cocatalyst layers that act as barriers to charge transport due to their poor conductivity. The resulting imbalance leads to localized charge accumulation and increases the recombination rate. Such dynamics are particularly pronounced in thicker CoFeO*_x_* layers, where the relative proportion of inactive layers increases, amplifying the impact of the recombination spikes registered in chopped voltammetries (Fig. 1A).

This interpretation is fully supported by the behavior of the thin cocatalyst 1 nm@BVO, where the majority of Co atoms sit at the CoFeO*_x_*/BVO interface. In this sample the $2_{\text{Ox}}^{D}$/$2_{\text{Red}}^{D}$ process below +1.0 V_RHE_ is the only one detected during the scans (Fig. 5C). Conversely, $1_{\text{Ox}}^{D}$/$1_{\text{Red}}^{D}$ appears neither in the $I_{\text{XRF}}$ nor in its derivative. In addition, no structural change associated to the latter is observed, as all Co sites are interfaced with the underlying BiVO_4_ layer. Consistently, the rate constants for recombination ( $k_{\text{rec}}$ ) and transfer ( $k_{\text{trans}}$ ) are respectively decreased and increased with respect to the thicker sample.

### Light-induced variations of Co oxidation state

The effect of sunlight illumination on the redox dynamics of cocatalysts in contact with the semiconductor, as revealed by Figs. 4 and 5, is twofold. We remark that the light-induced changes must be related to photoexcited charge carriers in the semiconductor, as the illumination has no effect on XANES/EXAFS in the case of 10 nm@FTO, in agreement with the previous conclusion that CoFeO*_x_* is not photoactive. On one side, the illumination provokes a shift toward lower potentials of the $2_{\text{Ox}}^{D}$/$2_{\text{Red}}^{D}$ process detected by FEXRAV, now named $2_{\text{Ox}}^{L}$/$2_{\text{Red}}^{L}$ (Fig. 5, B and C). This observation provides direct evidence that photogenerated holes in BVO are rapidly transferred to the cocatalyst (on the timescale of the FEXRAV scans), leading to an anticipated (retarded) Co oxidation during anodic (cathodic) scans. This interpretation also provides an explanation for the surface state capacitance revealed by P-EIS for CoFeO*_x_* (Fig. 1B) and for the decreased recombination rate determined by IMPS (Fig. 2). The transfer of holes from the semiconductor to the cocatalyst enhances their lifetime making the back recombination with CB electrons less probable. This results both in an increased chemical capacitance and a lower $k_{\text{rec}}$ . Moreover, the illumination increases the Co oxidation state at +0.2 V_RHE_ in 1 nm@BVO, in agreement with the picture of light-induced BVO-to-CoFeO*_x_* hole transfer at low potential. It is worthwhile to remind that the +0.5 to +1.0 V_RHE_ interval is the one where the cocatalyst brings about the most notable improvement of the PEC properties. Figure 4 also shows that, between +1.1 and +1.7 V_RHE_ and on the semiconductor only, the oxidation state under illumination does not change with the applied potential. Consistently, the $1_{\text{Ox}}^{D}$/$1_{\text{Red}}^{D}$ FEXRAV wave observed for 10 nm@BVO in the dark is suppressed under illumination. The above observations can be summarized by stating that, under illumination, the attainment of the highest oxidation state of Co atoms in contact with the semiconductor is shifted to more cathodic potentials, as a result of the photoexcited hole transfer.

On the other side, Fig. 4 shows that the Co oxidation state achieved under illumination is slightly lower compared to the dark case above ≈ 0.5 V. To explain this apparently contrasting behavior, it must be considered that under steady-state illumination a potential-independent accumulation of Co^II^ sites occurs in the cocatalyst compared to the dark case. Considerations on the rate-determining step (RDS) of the reaction, which affects the local structure and oxidation state of Co centers, may help in explaining why this is the case. For dark electrocatalysis on Co-based derivatives, the RDS was mostly identified as the O─O bond formation by a proton-coupled electron transfer mechanism involving water. This step typically involves a couple of Co^III^ sites, which promote O─O bond formation by producing either a peroxo or hydroperoxo intermediate (*41*, *42*). This is in contrast with the present evidence of a reduction process upon light excitation, suggesting a difference in RDS arising from the specific kinetics of photoinduced carriers transport across the semiconductor/catalyst interface. Such behavior is consistent with the formation of isolated Co^IV^ sites that undergo nucleophilic attack from water molecules, a process previously recognized by time-resolved Fourier transform infrared analysis (*43*) as a possible path for OER on Co-based catalysts. This path, which involves a step leading from Co^IV^ to Co^II^, is usually doomed by a low turnover frequency, compared to the cooperative formation of adjacent Co^IV^ sites and the following O─O bonding step. In conventional electrocatalytic experiments, where the kinetics of the charge transfer process is mainly limited by the transfer resistance of the cocatalyst, this results in a simultaneous large density of active Co^IV^ sites and increases the probability of having two neighboring Co^IV^ sites. On the other hand, in the photoinduced process, the number of photogenerated carriers available for the formation of active sites is limited by the generation/recombination dynamics, resulting in a lower density of active sites on the catalyst surface per unit time. This would favor the formation of “slow” catalytic sites, resting on the Co^II^ state until an additional photogenerated hole is injected. The average oxidation state is thus lowered by the presence of the photogenerated carriers, because more Co atoms will be in the Co^II^ state. As a possible alternative explanation, the cooperative role of Fe may be involved in the process. However, FEXRAV analysis on Fe K-edge does not indicate any change of the oxidation state of Fe sites upon illumination or external bias (fig. S14). It is also important to note that any potential temperature-induced effects on the redox properties of the cocatalyst, arising from photothermal heating of the catalyst, can be ruled out, as the observed temperature increase is minimal and insufficient to induce measurable changes in redox behavior (fig. S15).

## DISCUSSION

**Fig. 6.**
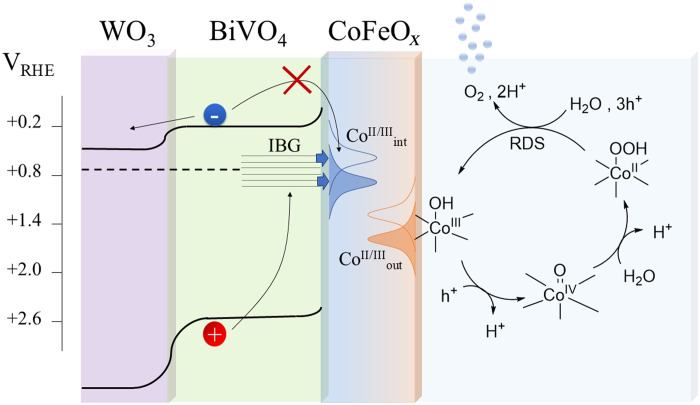
Mechanistic overview. Schematics of the interfacial charge transfer pathway for OER on BVO/CoFeO*_x_* photoanodes under sunlight irradiation. The gaussian distributions labelled ${\text{Co}}_{\text{out}}^{\text{II}/\text{III}}$ and ${\text{Co}}_{\text{int}}^{\text{II}/\text{III}}$ refer to the density of states function (*50*) for the redox transitions $1_{\text{Ox}}^{D}$/$1_{\text{Red}}^{D}$ and $2_{\text{Ox}}^{D}$/$2_{\text{Red}}^{D}$ , identified through FEXRAV analysis and associated with semiconductor/cocatalyst interface and cocatalyst/electrolyte interface, respectively.

The proposed catalytic cycle and the key findings from operando XAS characterization are summarized in Fig. 6. The interface-dependent Co^II/III^ redox couple, revealed by FEXRAV analysis, is shown to overlap with the surface trap states lying in the BVO semiconductor, as previously indicated by PEIS analysis of the samples with cocatalyst and dark CVs on bare BVO. The cathodic shift of the reversible wave observed in FEXRAV analysis under illumination suggests that the cocatalyst effectively scavenges holes trapped in the semiconductor surface states. This behavior enhances spatial charge separation, consistent with the increased Gartner current and reduced recombination rate in this potential range revealed by IMPS.

Furthermore, as demonstrated by FEXRAV and XANES analyses, no additional valency or structural change occur within the potential region where the photocurrent saturates. This implies that photoinduced hole trapping in the cocatalyst layer directly contributes to the OER turnover, despite the seemingly cathodic potential of the observed redox couple. Accumulation of charge carriers likely shifts the quasi-Fermi level of the semiconductor/cocatalyst system until the thermodynamic threshold for OER is met (*44*).

In conclusion, this study provides key insights into semiconductor/cocatalyst interactions in PEC water-splitting systems. The observed spontaneous charge transfer from the semiconductor to the cocatalyst and the identification of interface-specific reduction processes highlight the electronic coupling between the two components. The unusual behavior of Co oxidation states in the anodic region under illumination suggests the formation of slow catalytic sites upon photoexcitation, contrasting with conventional dark electrocatalysis. Specifically, isolated Co^IV^ sites reduce to Co^II^, leading to Co^II^ accumulation at the surface. The cocatalyst’s ability to exchange charge carriers with BiVO_4_ surface states under illumination, while mitigating recombination losses, underscores the necessity of understanding these dynamics under operando conditions.

Operando XAS analysis was instrumental in unraveling these intricate dynamic processes. Each operando technique contributed distinct and complementary insights into the photoelectrocatalytic behavior of the system: XANES mapped redox transitions and EXAFS resolved local structural distortions, while FEXRAV provided real-time redox voltammetry with element specificity. The latter revealed a reversible, interface-dependent redox mechanism, accompanied by a cathodic shift in redox potentials of the cocatalyst processes under illumination. These findings provide critical insights into the interplay between structural transformations in the cocatalyst and charge transfer at the semiconductor interface, directly demonstrating that photoexcited holes are scavenged by interfacial cobalt atoms. Moreover, the exceptional sensitivity and element-selectivity of FEXRAV elucidate the influence of cocatalyst thickness on redox behavior, offering a deeper understanding of the key factors governing PEC performance.

## MATERIALS AND METHODS

### Preparation of the heterojunction

Chemicals and solvents were purchased from Merck, Alfa Aesar, and Carlo Erba and were used as received. Water used for material preparations was deionized through a Millipore system.

#### FTO/WO_3_ electrodes

The preparation of spin-coated WO_3_ films constituted by WO_3_ nanoparticles was done according to previous literature (*45*, *46*). In brief, the preparation of colloidal WO_3_ was first accomplished by precipitating a H_2_WO_4_ gel by addition of concentrated HCl to aqueous Na_2_WO_4_ (Alfa Aesar, ≥ 99%). After several washings of H_2_WO_4_ carried out by redispersion/centrifugation, a stable H_2_WO_4_ sol was generated by peptization with oxalic acid (Alfa Aesar, ≥ 99.5%) at 60°C. The resulting colloidal suspension (having an overall mass of 8 to 8.5 g in the typical preparation) was densified by adding 20% (w/w) Carbowax (Sigma-Aldrich) and a few drops of Triton X-100 (Fluka), to improve the colloid spreadability during the subsequent FTO-coating process. Dry nanoparticulate films (colloidal photoanodes) were obtained onto the well-cleaned FTO glass (Pilkington TEC7) by spin coating the H_2_WO_4_ aqueous colloidal precursor described earlier. In the study herein reported, six WO_3_ layers were sequentially deposited, each layer being thermally annealed at 550°C for 30 min in air before the deposition of the next one. The total thickness of the electrode was 1 μm.

#### FTO/WO_3_/BiVO_4_ electrodes

Deposition of BiVO_4_ on WO_3_ photoanodes supported by FTO was achieved according to a previously published procedure (*47*), inspired by an electrochemical methodology developed by Seabold and Choi (*48*). In brief, 10 mM Bi(NO_3_)_3_ (Sigma Aldrich, ≥ 99%) was slowly added to an acidic solution of 10 mM VOSO_4_ (Alfa Aesar, 99.9%). Dropwise addition of concentrated HNO_3_ over a 30 min period was necessary to achieve the complete dissolution of Bi(NO_3_)_3_. After this point, the pH was rapidly increased to 4.5 using 2 M CH_3_COONa (Alfa Aesar, 99%). The resulting solution was quickly used as an electrolyte for two-electrode potentiostatic electrodeposition by applying 210 mV between FTO/WO_3_ and a platinum foil at room temperature for 300 s. The typical distance between the FTO/WO_3_ working electrode and the Pt foil was ≈ 3 mm. After deposition, the resulting FTO-supported photoanode was abundantly rinsed with water, dried at room temperature, and lastly annealed at 500°C in air for 2 hours.

#### FTO/WO_3_/BiVO_4_/CoFeO_x_

Deposition of the CoFeO*_x_* catalyst was performed according to Liardet *et al.* (*30*). This catalyst was deposited by chronoamperometry at 1.2 V versus saturated calomel electrode (SCE) for either 30 or 300 s under simulated AM 1.5G illumination. Reference FTO/CoFeO*_x_* was prepared with the same procedure, in absence of simulated sunlight.

### Morphological analysis

Morphological characterization was performed with a Zeiss Leo 1530 field-emission scanning electron microscope (FE-SEM), operated at 5 kV and equipped with in-lens secondary electrons detector. FE-SEM images of the photoanode surface and corresponding cross-sectional views are reported in fig. S1 for different deposition times of the CoFeO*_x_* overlayer. A thickness versus time calibration was determined from the linear fit of the CoFeO*_x_* thickness measured in cross section versus deposition time, as reported in fig. S2. However, because of the highly microporous structure, the thickness profile is highly inhomogeneous, and the nominal thickness must be considered as an estimate.

### PEC characterization

All PEC measurements have been performed in borate buffer 0.1 M solution at pH 9 with Na_2_SO_4_ 0.5 M as supporting electrolyte. The setup consisted in a PEEK custom flow cell connected to an AUTOLAB PGSTAT 204 Potentiostat. The reference electrode was an Ag/AgCl from ALS Japan and the counter electrode a Pt wire. All potentials were converted in volts versus reference hydrogen electrode (RHE) using the standard equation

$$V_{\text{RHE}}=V_{\text{Ag}/\text{AgCl}}+0.059\;\text{pH}+0.195$$

The IPCE were recorded in front illumination mode at 1.57 V_RHE_ and sampling rate of 1 s, by using an Xe source coupled to a monochromator. The value for the bare photoanode must be taken with some caution because of the limited stability of WO_3_/BiVO_4_ when nonprotected by the catalyst. UV-vis recorded in transmission mode against an FTO glass used as a reference. P-EIS under AM1.5 illumination was carried out with a 10 mV perturbation in the 50,000/0.05 Hz frequency range. Voltage was sampled at 20 mV intervals in the 0.6 to 1.12 V interval, which corresponds to the rising portion of the photocurrent in the *j*/*V* curve. After each sampled potential a *j*/*V* experiment was performed to verify the absence of electrode degradation. Fitting of P-EIS data was achieved with the equivalent circuit models outlined in fig. S5 (*31*). IMPS analysis was performed by illuminating with a UV light-emitting diode (385 nm) with 18 mW/cm^2^ DC intensity, adding an AC intensity modulation that had an RMS amplitude of 10% of the DC part. Chopped LSVs under the same DC light intensity are presented in fig. S7, exhibiting behavior closely resembling that observed under simulated sunlight conditions, with the exception of a different saturation current.

### Operando PEC-XAS setup

XAS characterization was performed to investigate the local structure of the cocatalyst thin layer. XAS experiments were conducted at the BM-08 LISA CRG Beamline (*49*) at the European Synchrotron Radiation Facility (ESRF) in Grenoble, in the course of the experimental sessions CH6417 and CH6747 (doi.org/10.15151/ESRF-ES-953228124 and doi.org/10.15151/ESRF-ES-1338642281). X-ray radiation is selected using a sagittal Si 111 double crystal monochromator (DCM), producing a beam size on the sample of approximately 100 μm × 100 μm. The energy resolution achieved with the Si 111 DCM is about $10^{-4}\Delta E/E$ . X-ray fluorescence intensity at the Co-Kα was registered with a 12-element High-Purity Ge detector and normalized to the incident x-ray beam intensity. Low-energy fluorescence from foreign elements, including substrate-related ones (Ca, Si, and Sn), was cut off by applying an Al filter on the fluorescence detector. Energy correction on Co reference was performed. Ex situ characterization was performed at room temperature in vacuum. The experimental apparatus for operando PEC measurements is discussed in the Supplementary Materials, including the main experimental parameters. The electrolyte used is borate buffer 0.1 M (pH 9), with the addition of 0.5 M Na_2_SO_4_ to minimize solution resistance. XAS spectra were registered at fixed potential in a three-electrode configuration, while FEXRAV is performed by performing a stepwise CV with scan rate 5 mV/s and step size 50 mV, synchronizing the x-ray detector sampling time to measure fluorescence intensity every 50 mV. Real-time correction for incident beam fluence was performed.

### EXAFS analysis

The XAS data analysis was carried out by Bruce Ravel’s Demeter software and Larch library. The fitting to a theoretical standard of EXAFS data was performed by FEFFIT. CoOOH-heterogeneite phase was used as reference for FEFF calculation of theoretical phases and amplitudes. The EXAFS spectra were modeled in the R-space from 1.0 to 3.5 Å, with a contribution of two single-scattering paths, corresponding to Co─O and Co─Co bonds. The Fourier transform interval was 3.0 to 8.0 Å^−1^. The fitting parameters were the energy shift Δ*E*, coordination numbers *N*, average interatomic distances *r*, and Debye-Waller factor σ^2^ for each path. Amplitude reduction factor was fixed to 1. FT-EXAFS spectra and related fits are reported in fig. S10, while fitting results are summarized in table S1. The Fe contribution was initially included as a substitutional dopant in the reference structure, but it was later neglected as the similar atomic radius to Co and the limited *k* range of the experimental data resulted in poor fitting reliability.

## Supplementary Materials

### This PDF file includes:

Supplementary Text Figs. S1 to S15 Table S1 References
