# Supplementary material for: Operando x-ray absorption spectroscopy unveils light-driven redox dynamics at the semiconductor/cocatalyst interface
Source: Sci Adv. 2025 Sep 19;11(38):eadx8089. doi: 10.1126/sciadv.adx8089 (PMC12448062; doi:10.1126/sciadv.adx8089)
Supplement: Supplementary file 1 — Supplementary Text Figs. S1 to S15 Table S1 References [file sciadv.adx8089_sm.pdf]

Science Advances
AAAS

Supplementary Materials for

Operando x-ray absorption spectroscopy unveils light-driven redox dynamics at the semiconductor/cocatalyst interface

Raffaello Mazzaro *et al.*

Corresponding author: Raffaello Mazzaro, raffaello.mazzaro@unibo.it

Sci. Adv. **11**, eadx8089 (2025)
DOI: 10.1126/sciadv.adx8089

This PDF file includes:

Supplementary Text
Figs. S1 to S15
Table S1
References

Supplementary text

Cyclic Voltammetry

Figure S3b shows the dark cyclic voltammetries (CVs) performed with a slow scan rate of 5 mV/s, the same employed for the FEXRAV measurements. In *10nm@BVO*, two overlapping anodic waves around 1.37 and 1.45 V are visible before the onset of the OER current, counteracted by a broader wave in the cathodic scan centered at about 1.3 V. The absence of these waves in the bare photoanode indicates that they mark a change in oxidation state of atoms in the co-catalyst layer, and in fact their average potential agrees with the one reported in literature for the $Co^{II/III}$ and $Co^{III/IV}$ transitions (*15*), respectively. Consistently, in the *1nm@BVO* photoanode the anodic wave is still visible although very weak, while the broad reductive wave is not detected due to the extremely thin cocatalyst layer and the overlapping capacitive current of the highly porous material.
Furthermore, *10nm@BVO* shows a cathodic wave in the 1.0 → 0.6 V scan interval, which is not detected in the other photoanodes and can therefore be tentatively ascribed to a second reduction process specific of the $CoFeO_x$ overlayer. The FEXRAV analysis will show that this is indeed the case and reveal the same process on the thin overlayer thanks to its element-specific sensitivity.
Finally, a reduction wave is detected on all samples in the 0.6 → 0.4 V scan interval, which has previously been ascribed to the filling of shallow intra band gap states of $BiVO_4$ corresponding to the passage of vanadium from the V^{V} state to the V^{IV} one. This wave is superimposed on a large cathodic current, which has been observed also in WO_3 thin films without $BiVO_4$ and has been attributed to the $W^{VI/V}$ reduction (*23*).

Incident Photon to Electron (IPCE) and UV-Vis spectroscopy

The strong reduction of IPCE values for the 10nm@BVO sample is due to poor charge transport and decreased optical transmittance across the thick co-catalyst overlayer. This is confirmed by the UV-Vis spectra. Indeed, Figure S4b clearly shows that the thick co-catalyst overlayer enhances the absorption in the 500-400 nm range, thus lowering the photon flux on the $WO_3/BiVO_4$ heterojunction. Overall, the UV-Vis spectra represent a combination of the absorption spectra of WO_3 and $BiVO_4$, with the main absorption occurring in the 400-450 nm range.

Photoelectrochemical Impedance Spectroscopy (P-EIS)

Representative Nyquist plots as a function of the applied voltage are reported in Figure S5, showing that the equivalent circuits proposed achieve a satisfactory fitting of the experimental points, with relative errors < 10%. The models chosen are different depending on the thickness of the $CoFeO_x$ film. For *1nm@BVO* the data were fitted using two serially connected RC meshes: R2/CPE1 that describes the FTO/WO_3 interface and the nested mesh where R3/CPE2 models the charge transfer across the $WO_3/BiVO_4$ interface with an additional charge transfer layer (R4/CPE3) describing the transfer through the CoFeOx overlayer.
For the *10nm@BVO* photoanode, fitting was performed using only two serially connected RC meshes, where R2/CPE1 describes the FTO/WO_3 interface as before, while the second (R3/CPE2) models the $CoFeO_x$/electrolyte interface. In the presence of a thick $CoFeO_x$ overlayer, the nested circuit resulted in large errors and small semiconductor capacitance, indicating that the interfacial

charge transfer process is dominated by charge transport through the thick co-catalyst wherein the photogenerated holes are transferred. This is consistent with the strong recombination features observed in the chopped j/V curves (Figure 1a).
Non-ideal capacitances are modelled as constant phase elements (CPE) according to:

$$Z_{CPE} = \frac{1}{T \times \omega^{n-1}} \quad (S1)$$

The equivalent capacitance was obtained using the formula (*51*):

$$C = T^{\frac{1}{n}} \times R_{CT}^{\frac{1-n}{n}} \quad (S2)$$

Where T is the CPE admittance, n is the CPE exponent and R_{CT} is the parallel charge transfer resistance.

The equivalent circuits describe well the j/V characteristics of the photoanodes, as shown by the good correlation of the real part of the total impedance with the reciprocal derivative of the j/V curve. (Figure S6).

Intensity Modulated Photocurrent Spectroscopy (IMPS)

For the IMPS measurements, light was provided through a UV LED (385 nm) with 18 mW/cm^2 DC intensity, adding an AC intensity modulation that had an RMS amplitude of 10% of the DC part. Chopped LSVs under the same DC light intensity are presented in Fig. S7, exhibiting behavior closely resembling that observed under simulated sunlight conditions, with the exception of a different saturation current.

Operando PEC-XAS - Experimental setup description

In order to perform operando measurements, we developed an experimental setup fully integrated with the beamline, which allows to control the main experimental parameters directly from the control room. The scheme is reported in Figure S9. The potential applied to the cell and the resulting current can be monitored with the potentiostat, feeding this data directly to the acquisition system of the beamline for a precise synchronization between electrochemical and fluorescence data. A compact light source based on a high-power white LED was placed into the chamber, allowing the illumination of the photoelectrode either from the back or the front (through the electrolyte and the Kapton window). The emission spectrum of the LED is reported in Figure S9c. The emission power of the LED was tuned to obtain the equivalent illumination of 1 sun (100mW/cm^2).

The design of the PEC cell (Figure S9b) used for operando measurements was inspired by previous works (*21,52*) and re-designed by us to meet our experimental needs, arising from the type of measurement, the type of sample, and the specific beamline. The key features of our PEC cell are:

- It is 3D printed (we can share the CAD file if needed);
- The substrates on which the samples are deposited do not need to be tailored for exclusive use with the operando PEC cell, and substrates of various thicknesses and sizes (from a minimum of 10 × 15 mm to a maximum of 40 × 40 mm);

- Reduced electrolyte thickness (~100 μm) in front of the sample to prevent X-ray signal attenuation;
- Channeled electrolyte flow above the sample for more efficient bubble removal.

In addition, thanks to the use of a pulseless 3D printed peristaltic pump, the flux of the electrolyte in the cell can be controlled for enhancing mass transport and removing gaseous product from the electrode's surface.

The cell was attached to the motorized stage of the chamber (Figure S9b, left) usually employed for samples positioning, allowing to move it and change the measurement spot to avoid X-ray damage during long measurements. The synchronization between the potentiostat, the light source and the peristaltic pump was ensured by an Arduino microcontroller and a custom LabVIEW control program. The main chamber can be both put under vacuum or filled with helium gas for reducing X-ray attenuation.

Extended X-ray Fine Structure (EXAFS)

The results of EXAFS analysis are reported in the following. It must be noted that in 1nm@BVO the second coordination shell contribution to the EXAFS is almost fully damped in vacuum, as indicated by the lack of a second peak in the FT-EXAFS (Fig S10, Table S1 data). Consistently, the coordination number for the Co-Co scattering path is lower than 1, pointing out a highly disordered structure. However, a second peak appears upon equilibration with the electrolyte, with $N_{Co\text{-}Co}$ and $r_{Co\text{-}O}$ conforming with the values observed for thicker catalyst layers.

Fixed Energy X-Ray Absorption Voltammetry (FEXRAV)

Fixed Energy X-ray voltammetry was performed at BM08 (LISA) beamline at ESRF, Grenoble. Fig S13 (red line) shows the fluorescence signal normalized with the incident beam at a fixed photon energy, corresponding to the maximum derivative of the absorption spectrum (7723 eV) recorded at open circuit potential. The orange plot corresponds to the opposite of the derivative of the FEXRAV signal. The blue plot is the current recorded during the experiment and the grey plot is the potential applied to the cell.

Supplementary Figures

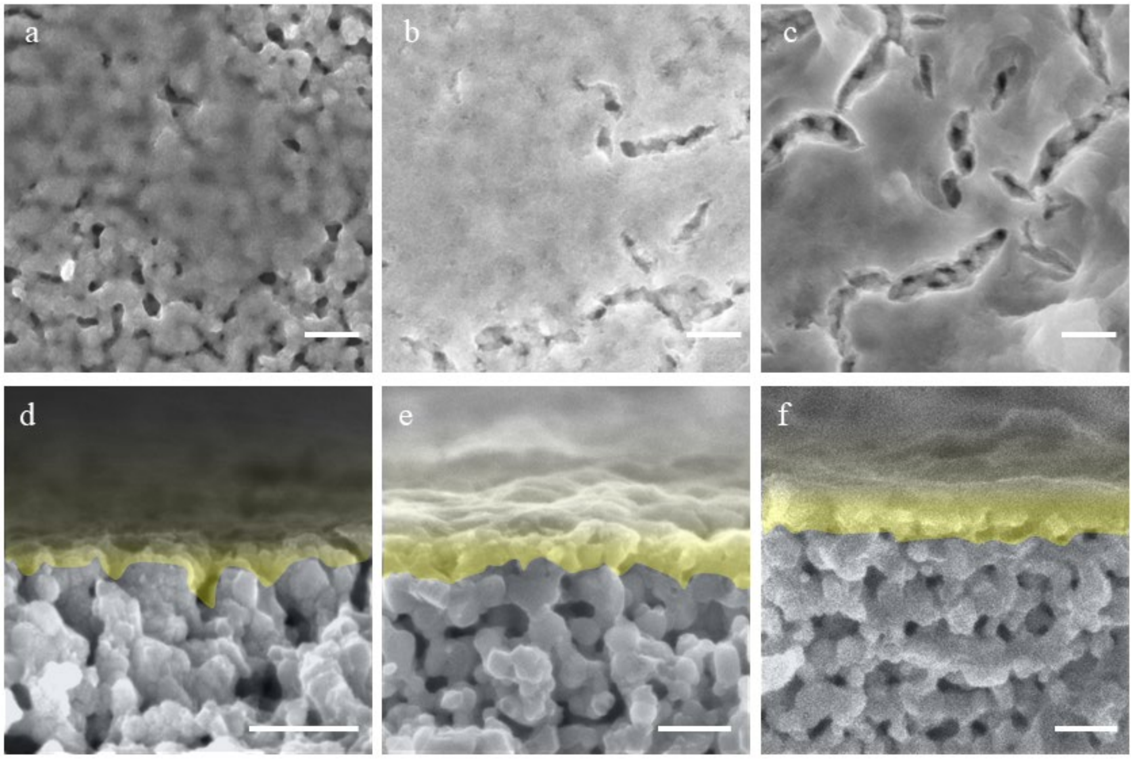

Figure S1. Morphological characterization: SEM micrographs of $WO_3/BiVO_4$ with increasing $CoFeO_x$ deposition time, namely a) 300s, b) 1200s and c) 1800s. d,e,f) Corresponding cross-sectional views highlighting the COFeOx layer (yellow). Scale bar 200nm.

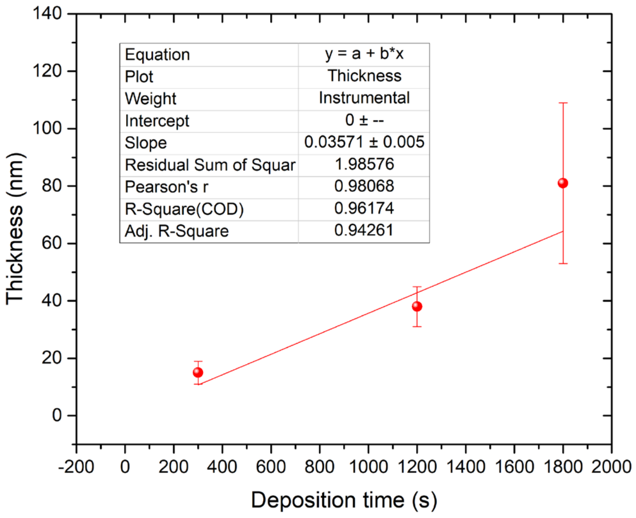

Figure S2. CoFeOx Thickness estimation: Linear fit of the $CoFeO_x$ overlayer thickness, as determined from FE-SEM cross-sections, vs deposition time.

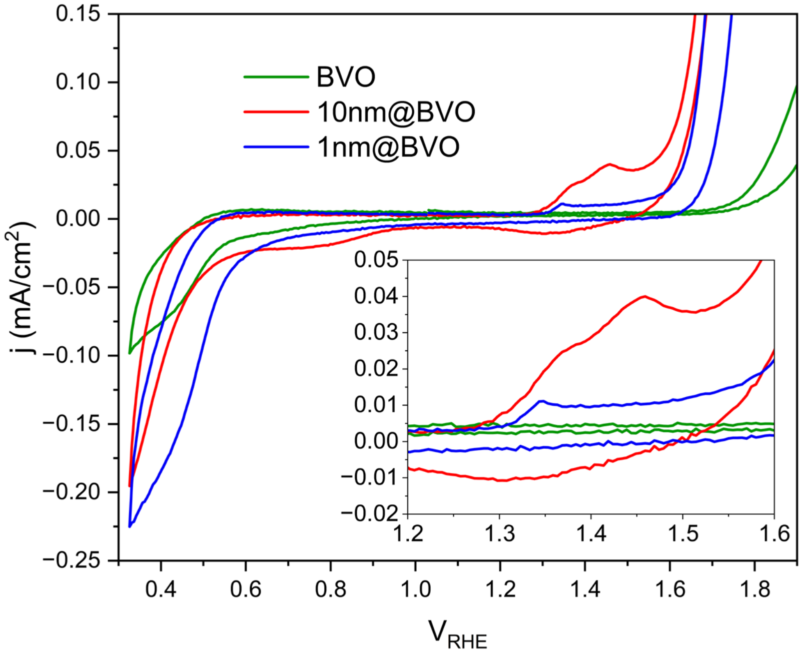

Figure S3. Dark electrochemical characterization: Cyclic voltammetries at 5 mV/s for the three photoanodes.

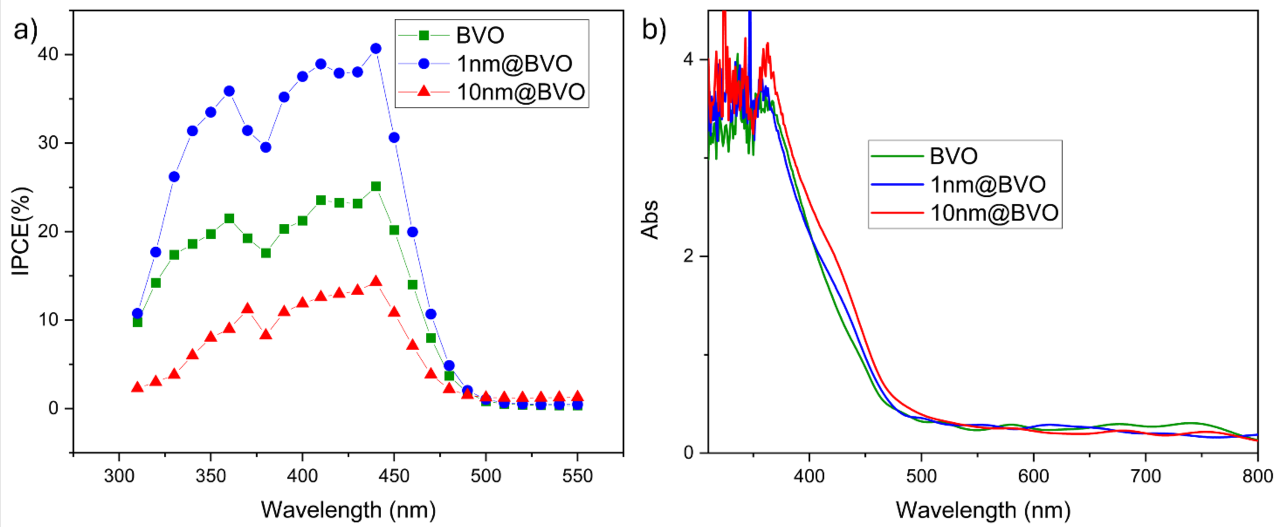

Figure S4. Spectral efficiency: a) IPCE spectra recorded at 1.57 V_{RHE} and b) UV-Vis spectra.

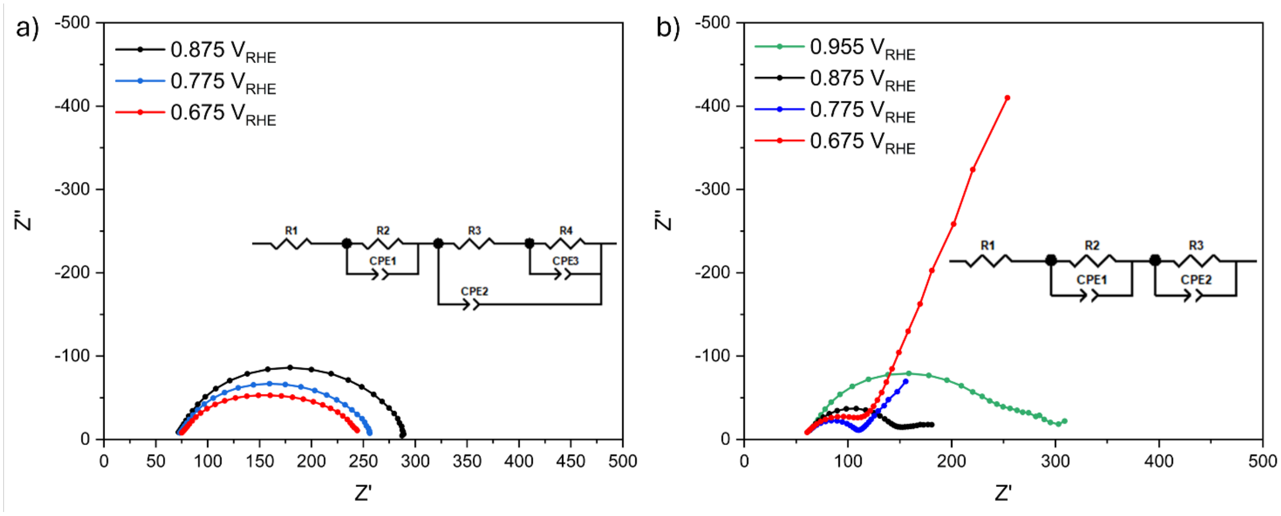

Figure S5. Electrochemical impedance: P-EIS Nyquist plots and respective equivalent circuit models for a) *1nm@BVO* (deposition time=30 s) and b) 1*0nm@BVO* (deposition time=300 s).

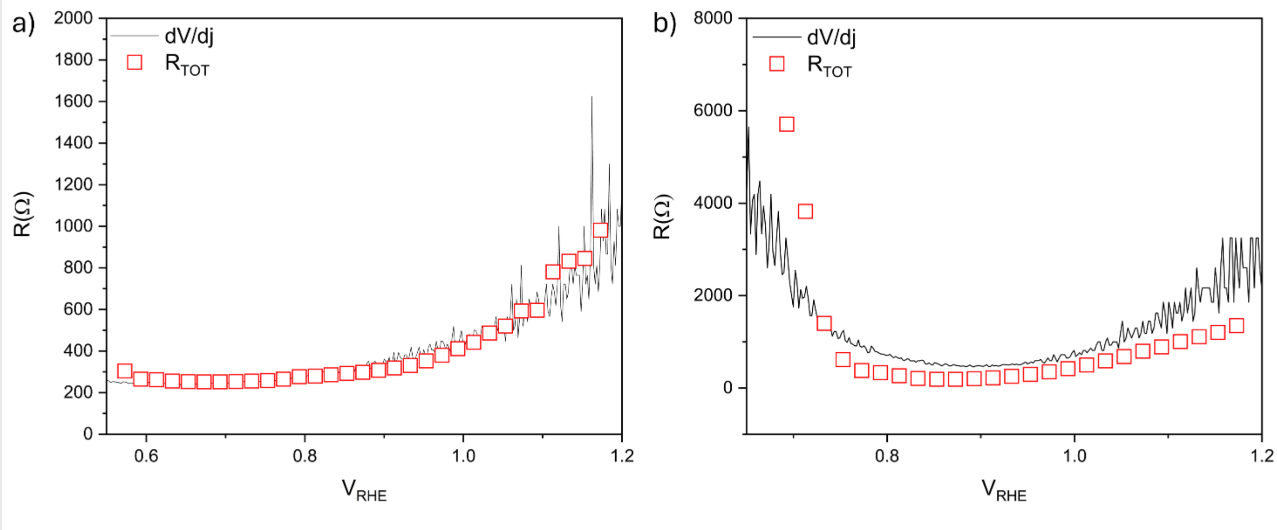

Figure S6. Electrochemical impedance: R vs $\partial V/\partial j$ in a) *1nm@BVO* (deposition time=30 s) and b) *10nm@BVO* (deposition time=300 s).

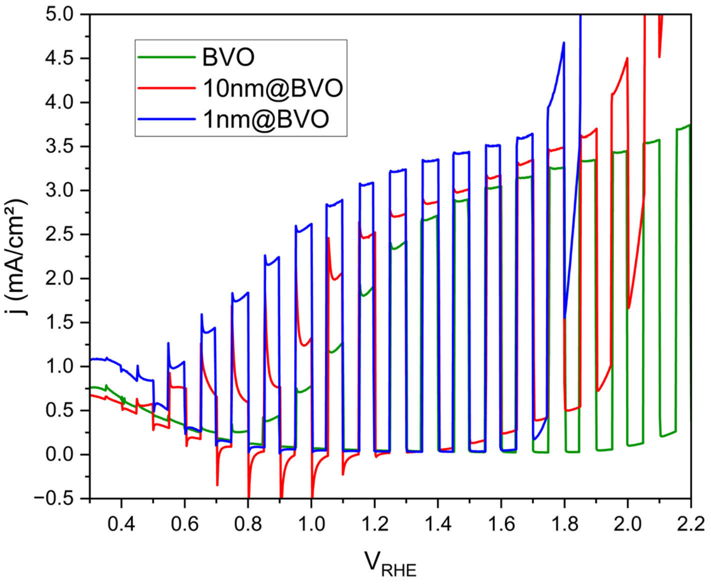

Figure S7. Monochromatic chopped voltammetries: Chopped light linear sweep voltammetries with the same DC light conditions of IMPS.

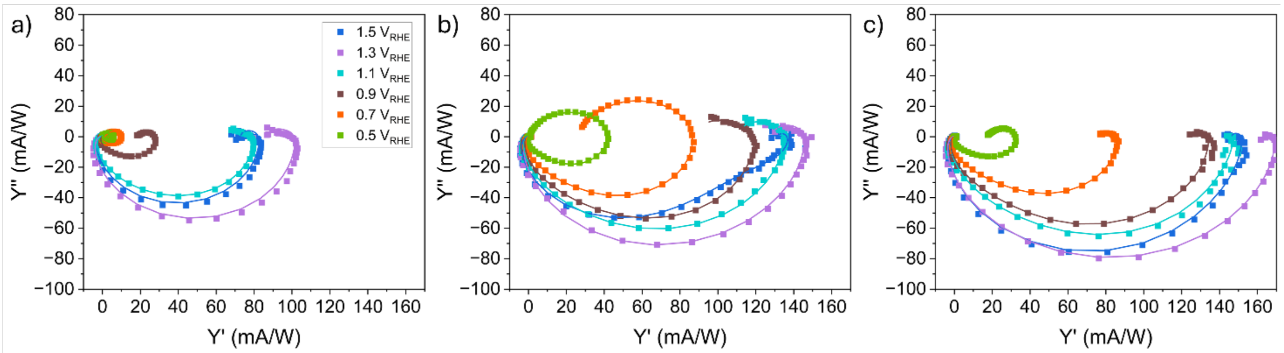

Figure S8. IMPS analysis: IMPS measurements for the three samples (squares) at different potentials and corresponding fit with DRT Lasso regolarization algorithm (solid lines): a) bare *$WO_3/BiVO_4$*; b) *1nm@BVO* and c) *10nm@BVO*. Legend is showed only for a) but colors are the same across the three pictures.

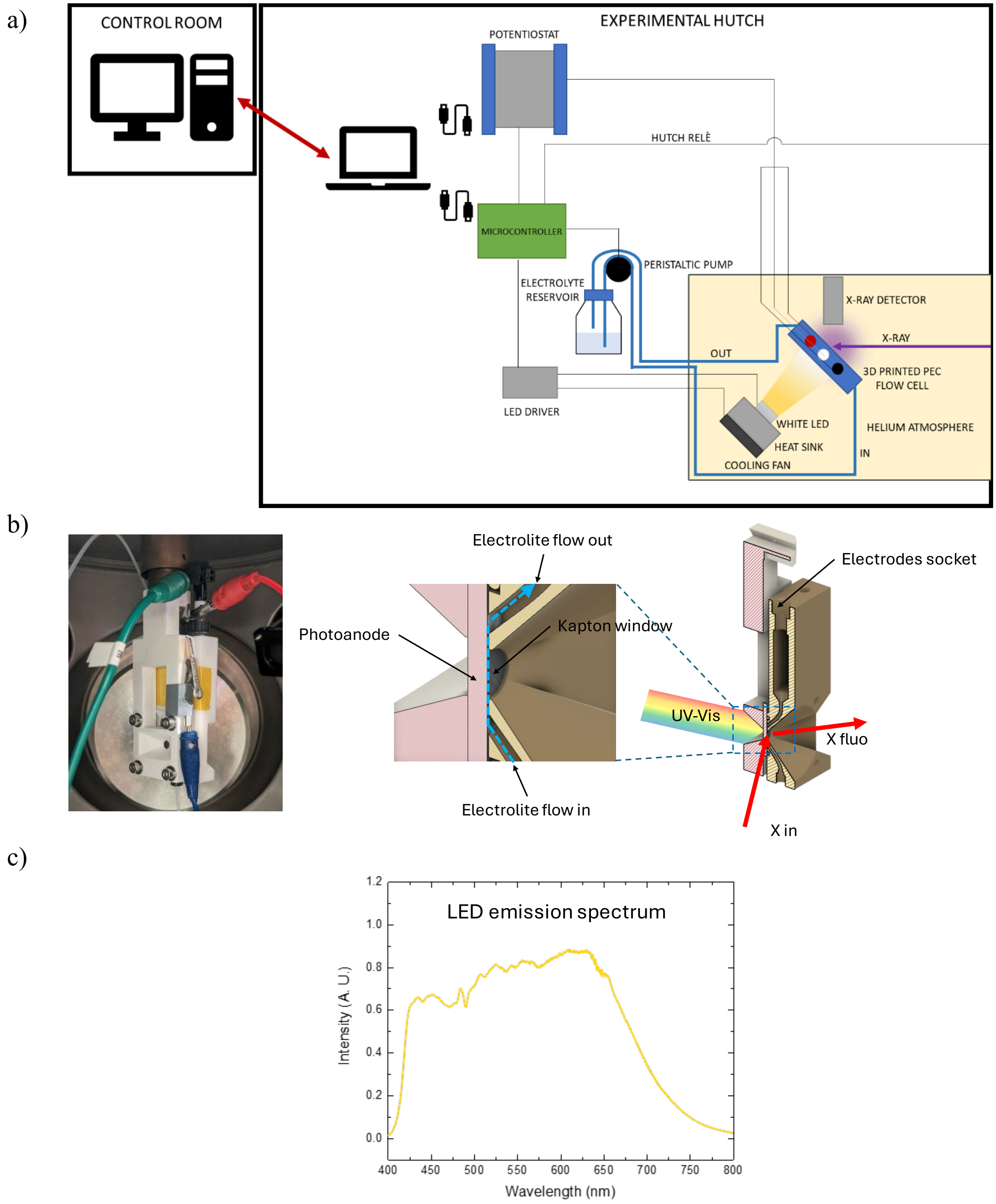

Figure S9. Operando PEC-XAS setup: Experimental setup developed for operando X-ray absorption measurements integrated with LISA beamline at ESRF, Grenoble. (a) Scheme of the experimental setup with all the main components: potentiostat, microcontroller for pulseless peristaltic pump, electrolyte reservoir, 3D printed cell and white LED. (b) Design of the cell: left – picture of the cell in the vacuum chamber, right – cross-section of the cell. (c) Emission spectrum of the white LED used for operando measurements.

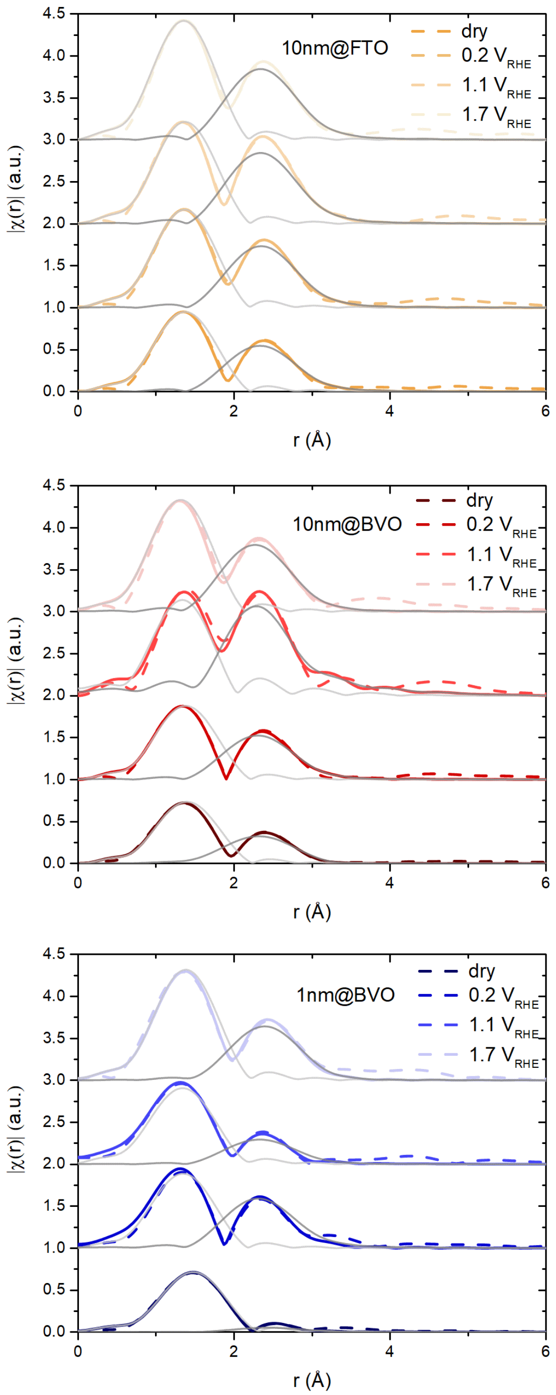

Figure S10. EXAFS data fitting: FT-EXAFS data (dashed lines) for (top) 1nm@BVO, (center) 10nm@BVO and (bottom) 10nm@FTO at selected potentials vs RHE. Fitting results for single scattering Co-O and Co-Co paths (grey lines) and resulting composite fitting (coloured continuous lines) are overlayed. The same plots are reported also for dry samples for comparison purposes.

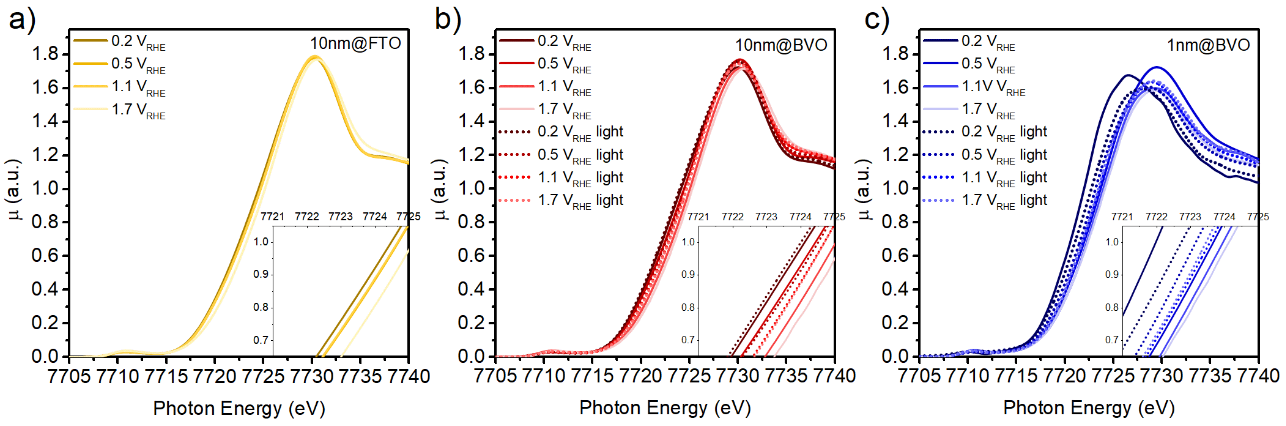

Figure S11. XANES analysis - Co K-edge XANES of a) *1nm@BVO*, b) *10nm@BVO*, and c)*10nm@FTO* measured at selected potentials, in dark (solid lines) and under AM 1.5G irradiation (dotted lines). In the inset, zoomed view of the potential- and light- dependent edge shift.

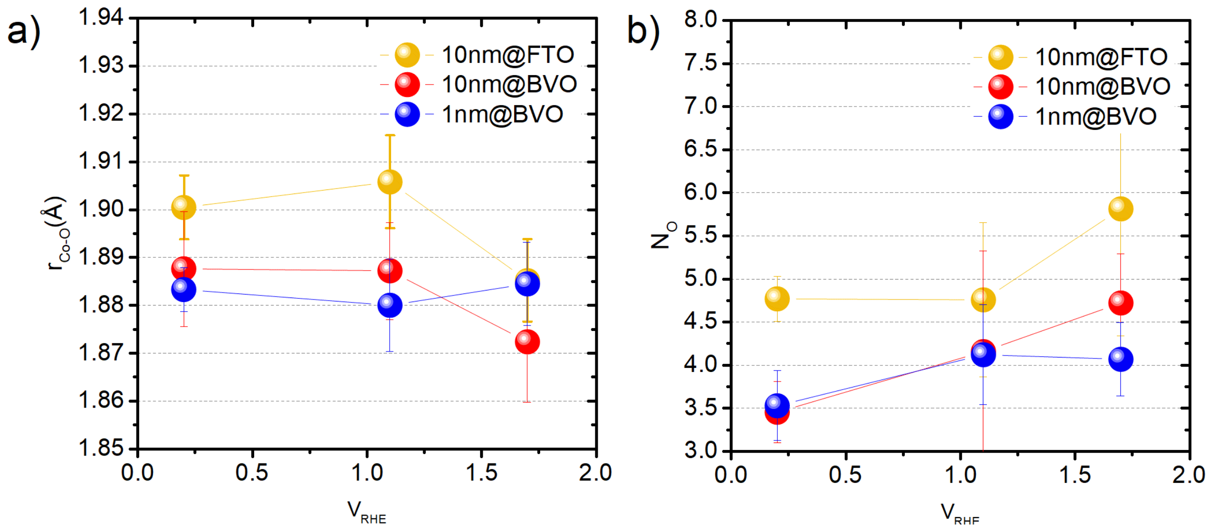

Figure S12. EXAFS data analysis: – a) Co–O interatomic distance and b) coordination number determined from fitting the first Co–O coordination shell in FT-EXAFS at selected applied potentials in the dark. The error bars for the coordination numbers and Co–O distances are provided by the standard EXAFS fitting procedure.

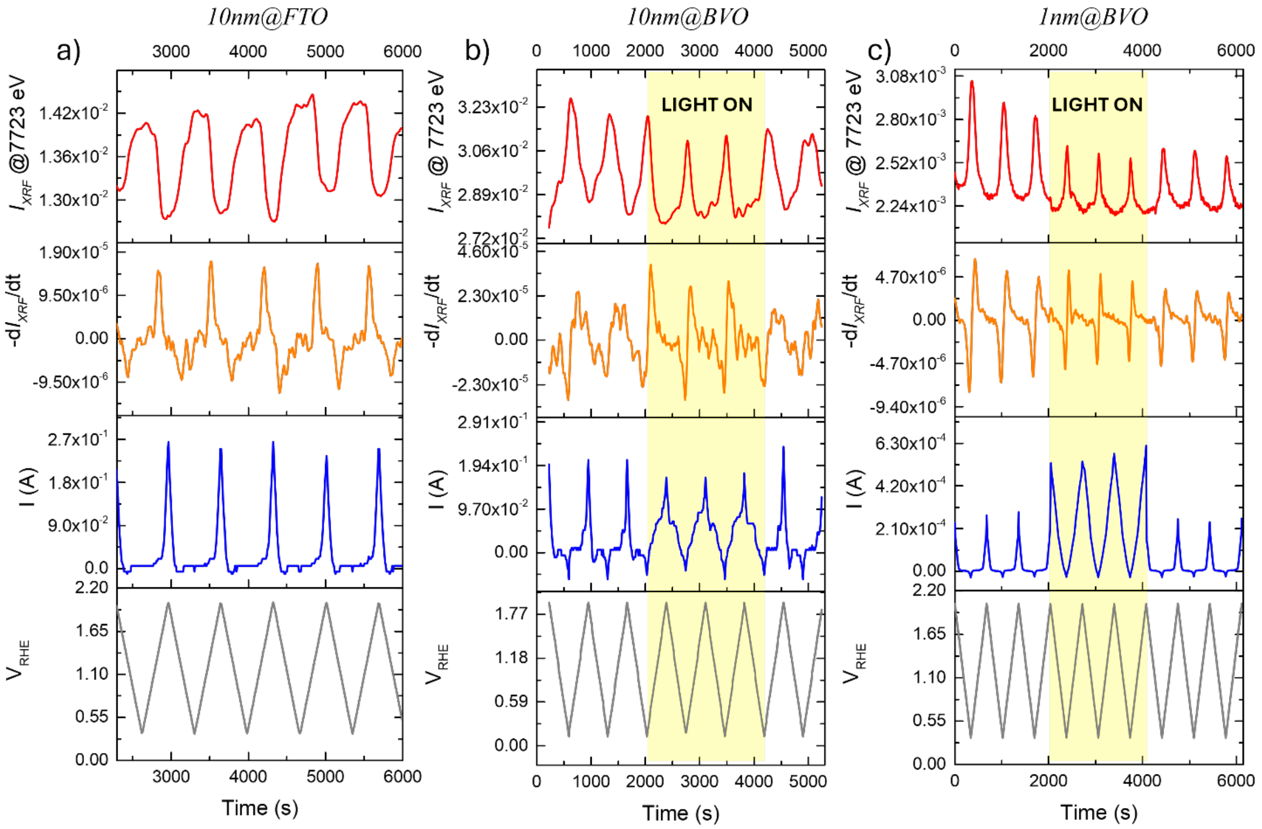

Figure S13. FEXRAV raw data: FEXRAV data as a function of time: fluorescence signal normalized to the incident beam at a fixed photon energy (I_{XRF}), corresponding to the maximum derivative of the absorption spectrum recorded at open circuit potential (red line), its derivative ($-dI_{XRF}/dt$, orange), current measured during the experiment (blue), potential applied to the cell (grey). Several cycles were performed in order to confirm the stability of the sample and the reproducibility of the results. White LED light was also turned on during some cycles to simulate sun illumination.

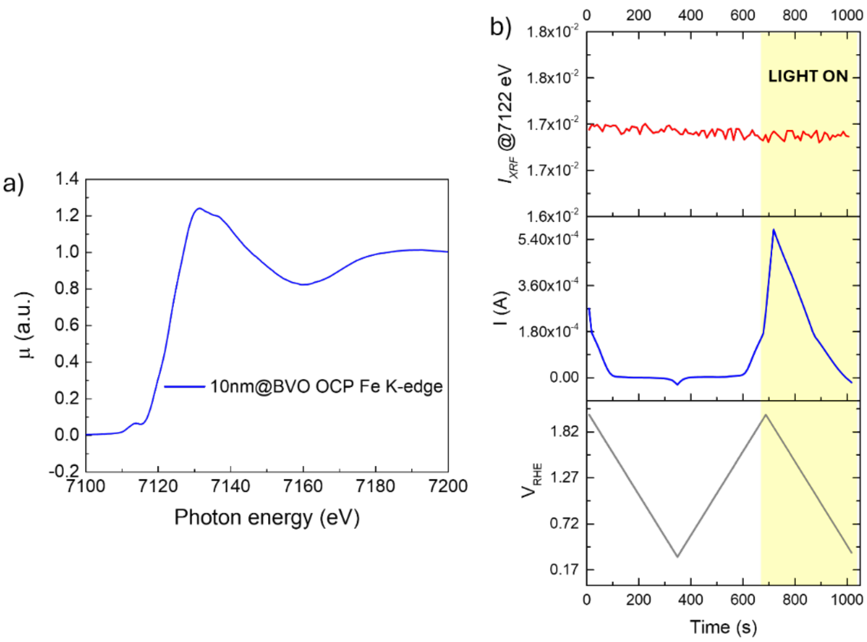

Figure S14. XAS analysis at Fe K-edge: a) XANES spectra at Fe K-edge for *10nm@BVO* sample. b) FEXRAV in function of time at the Fe K-edge: fluorescence signal normalized to the incident beam at a fixed photon energy, corresponding to the maximum derivative of the absorption spectrum recorded at open circuit potential (red line), current measured during the experiment (blue), potential applied to the cell (grey).

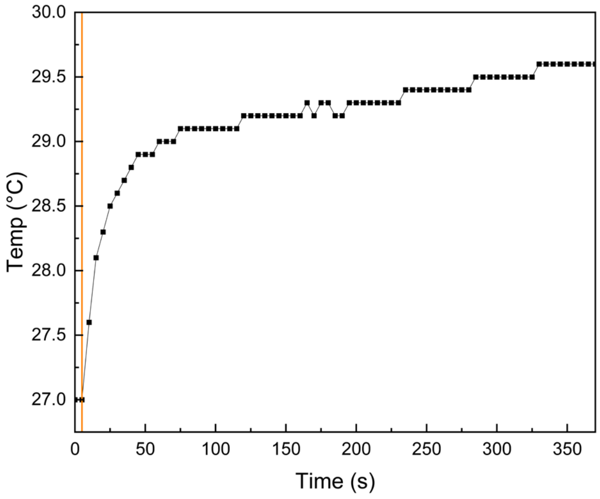

Figure S15. Photothermal activity: Temperature variation at the electrode surface during operando XAS measurements. The yellow line indicates the onset of illumination. A maximum temperature increase of approximately 2.5 °C is observed after 350 s. Temperature was recorded using a thermocouple in direct contact with the Kapton window of the operando-XAS cell. Given the limited thickness of the electrolyte layer, the measured temperature closely approximates that of the electrode surface.

Supplementary tables

Table S1. Results of fitting EXAFS data.

	ΔE_0 (eV)	N_O	ΔN_O	r_{Co-O} (Å)	Δr (Å)	$\sigma^2(O)$	$\Delta\sigma$	N_{Co}	ΔN_{Co}	r_{Co-Co} (Å)	Δr (Å)	$\sigma^2(Co)$	$\Delta\sigma$	R-fac. (%)
**10nm@FTO dry**	-3.32	3.66	0.28	1.901	0.010	0.001	0.013	6.78	1.45	2.849	0.017	0.006	0.006	4.1
**10nm@FTO +0.2V_{RHE}**	-4.34	4.75	0.26	1.900	0.006	0.001	0.002	8.71	1.75	2.864	0.019	0.005	0.005	2.2
**10nm@FTO +1.1 V_{RHE}**	-3.78	4.75	0.89	1.905	0.009	0.001	0.006	11.1	1.69	2.850	0.013	0.006	0.004	4.3
**10nm@FTO +1.7 V_{RHE}**	-4.22	5.81	1.47	1.885	0.008	0.002	0.010	9.56	2.04	2.857	0.023	0.004	0.006	2.1
**10nm@BVO dry**	-2.88	2.77	0.13	1.923	0.010	0.003	0.004	8.52	2.11	2.863	0.010	0.024	0.005	2.2
**10nm@BVO +0.2 V_{RHE}**	-4.21	3.45	0.35	1.887	0.012	0.002	0.001	8.32	2.91	2.827	0.018	0.007	0.008	3.8
**10nm@BVO +1.1 V_{RHE}**	-5.01	4.16	1.17	1.887	0.010	0.001	0.019	10.22	2.03	2.836	0.034	0.001	0.006	5.6
**10nm@BVO +1.7 V_{RHE}**	-5.05	4.72	0.57	1.872	0.012	0.001	0.000	11.23	3.25	2.821	0.031	0.007	0.008	6.7
**1nm@BVO dry**	-0.22	4.33	0.24	2.033	0.004	0.003	0.008	0.43	0.85	2.959	0.035	0.009	0.027	9.9
**1nm@BVO +0.2 V_{RHE}**	-5.27	3.52	0.41	1.883	0.004	0.002	0.001	3.58	1.57	2.820	0.022	0.001	0.017	7.0
**1nm@BVO +1.1 V_{RHE}**	-5.09	4.12	0.58	1.880	0.009	0.004	0.001	2.58	1.75	2.839	0.024	0.007	0.010	6.6
**1nm@BVO +1.7 V_{RHE}**	-5.01	4.07	0.42	1.884	0.008	0.001	0.007	3.81	2.84	2.901	0.021	0.012	0.006	7.6

REFERENCES AND NOTES

1. T. Gatti, F. Lamberti, R. Mazzaro, I. Kriegel, D. Schlettwein, F. Enrichi, N. Lago, E. Di Maria, G. Meneghesso, A. Vomiero, S. Gross, Opportunities from doping of non-critical metal oxides in last generation light-conversion devices. *Adv. Energy Mater.* **11**, 2101041 (2021).

2. B. Moss, F. S. Hegner, S. Corby, S. Selim, L. Francàs, N. López, S. Giménez, J.-R. Galán-Mascarós, J. R. Durrant, Unraveling charge transfer in CoFe prussian blue modified $BiVO_4$ photoanodes. *ACS Energy Lett.* **4**, 337–342 (2019).

3. E. Fabbri, D. F. Abbott, M. Nachtegaal, T. J. Schmidt, *Operando* X-ray absorption spectroscopy: A powerful tool toward water splitting catalyst development. *Curr. Opin. Electrochem.* **5**, 20–26 (2017).

4. M. Fracchia, P. Ghigna, A. Vertova, S. Rondinini, A. Minguzzi, Time-resolved X-ray absorption spectroscopy in (photo)electrochemistry. *Surfaces* **1**, 138–150 (2018).

5. R. Yalavarthi, O. Henrotte, A. Minguzzi, P. Ghigna, D. A. Grave, A. Naldoni, In situ characterizations of photoelectrochemical cells for solar fuels and chemicals. *MRS Energy Sustain.* **7**, E37 (2020).

6. A. Minguzzi, P. Ghigna, *X-Ray Absorption Spectroscopy in Electrochemistry from Fundamentals to Fixed Energy X-Ray Absorption Voltammetry* (CRC Press-Taylor and Francis Group: Boca Raton, FL, USA, 2017).

7. D. F. Abbott, D. Lebedev, K. Waltar, M. Povia, M. Nachtegaal, E. Fabbri, C. Copéret, T. J. Schmidt, Iridium oxide for the oxygen evolution reaction: Correlation between particle size, morphology, and the surface hydroxo layer from operando XAS. *Chem. Mater.* **28**, 6591–6604 (2016).

8. V. Pfeifer, T. E. Jones, J. J. Velasco Vélez, R. Arrigo, S. Piccinin, M. Hävecker, A. Knop-Gericke, R. Schlögl, In situ observation of reactive oxygen species forming on oxygen-evolving iridium surfaces. *Chem. Sci.* **8**, 2143–2149 (2017).

9. M. Görlin, P. Chernev, J. F. De Araújo, T. Reier, S. Dresp, B. Paul, R. Krähnert, H. Dau, P. Strasser, Oxygen evolution reaction dynamics, faradaic charge efficiency, and the active metal redox states of Ni-Fe oxide water splitting electrocatalysts. *J. Am. Chem. Soc.* **138**, 5603–5614 (2016).

10. T. Y. Lai, Y. C. Chu, Y. A. Lai, C. J. Chang, C. H. Wu, Y. Y. Chen, C. Y. Liu, D. Y. Wu, C. W. Tung, H. M. Chen, Operando identification of synergistic dynamic for cobalt-tungsten-based bimetallic electrocatalysts in oxygen evolution reaction. *Mater Today Sustain* **24**, 100487 (2023).

11. R. D. L. Smith, C. Pasquini, S. Loos, P. Chernev, K. Klingan, P. Kubella, M. R. Mohammadi, D. Gonzalez-Flores, H. Dau, Spectroscopic identification of active sites for the oxygen evolution reaction on iron-cobalt oxides. *Nat. Commun.* **8**, 2022 (2017).

12. N. Li, R. G. Hadt, D. Hayes, L. X. Chen, D. G. Nocera, Detection of high-valent iron species in alloyed oxidic cobaltates for catalysing the oxygen evolution reaction. *Nat. Commun.* **12**, 6–11 (2021).

13. L. J. Enman, M. B. Stevens, M. H. Dahan, M. R. Nellist, M. C. Toroker, S. W. Boettcher, Operando X-ray absorption spectroscopy shows iron oxidation is concurrent with oxygen evolution in cobalt–iron (oxy)hydroxide electrocatalysts. *Angew. Chem. Int. Ed.* **57**, 12840–12844 (2018).

14. M. S. Burke, M. G. Kast, L. Trotochaud, A. M. Smith, S. W. Boettcher, Cobalt-iron (oxy) hydroxide oxygen evolution electrocatalysts: The role of structure and composition on activity, stability, and mechanism. *J. Am. Chem. Soc.* **137**, 3638–3648 (2015).

15. H.-Y. Wang, S.-F. Hung, H.-Y. Chen, T.-S. Chan, H. M. Chen, B. Liu, In operando identification of geometrical-site-dependent water oxidation activity of spinel Co_3O_4. *J. Am. Chem. Soc.* **138**, 36–39 (2016).

16. J. T. Mefford, A. R. Akbashev, M. Kang, C. L. Bentley, W. E. Gent, H. D. Deng, D. H. Alsem, Y. Yu, N. J. Salmon, D. A. Shapiro, P. R. Unwin, W. C. Chueh, Correlative operando microscopy of oxygen evolution electrocatalysts. *Nature* **593**, 67–73 (2021).

17. M. Risch, F. Ringleb, M. Kohlhoff, P. Bogdanoff, P. Chernev, I. Zaharieva, H. Dau, Water oxidation by amorphous cobalt-based oxides: In situ tracking of redox transitions and mode of catalysis. *Energ. Environ. Sci.* **8**, 661–674 (2015).

18. M. W. Kanan, J. Yano, Y. Surendranath, M. Dincă, V. K. Yachandra, D. G. Nocera, Structure and valency of a cobalt-phosphate water oxidation catalyst determined by in situ X-ray spectroscopy. *J. Am. Chem. Soc.* **132**, 13692–13701 (2010).

19. D. Friebel, M. Bajdich, B. S. Yeo, M. W. Louie, D. J. Miller, H. Sanchez Casalongue, F. Mbuga, T.-C. Weng, D. Nordlund, D. Sokaras, R. Alonso-Mori, A. T. Bell, A. Nilsson, On the chemical state of Co oxide electrocatalysts during alkaline water splitting. *Phys. Chem. Chem. Phys.* **15**, 17460–17467 (2013).

20. N. Li, D. K. Bediako, R. G. Hadt, D. Hayes, T. J. Kempa, F. Von Cube, D. C. Bell, L. X. Chen, D. G. Nocera, Influence of iron doping on tetravalent nickel content in catalytic oxygen evolving films. *Proc. Natl. Acad. Sci. U.S.A.* **114**, 1486–1491 (2017).

21. E. Achilli, A. Minguzzi, A. Visibile, C. Locatelli, A. Vertova, A. Naldoni, S. Rondinini, F. Auricchio, S. Marconi, M. Fracchia, P. Ghigna, 3D-printed photo-spectroelectrochemical devices for in situ and in operando X-ray absorption spectroscopy investigation. *J. Synchrotron Radiat.* **23**, 622–628 (2016).

22. A. Braun, K. Sivula, D. K. Bora, J. Zhu, L. Zhang, M. Grätzel, J. Guo, E. C. Constable, Direct observation of two electron holes in a hematite photoanode during photoelectrochemical water splitting. *J. Phys. Chem. C* **116**, 16870–16875 (2012).

23. M. Fracchia, V. Cristino, A. Vertova, S. Rondinini, S. Caramori, P. Ghigna, A. Minguzzi, Operando X-ray absorption spectroscopy of WO_3 photoanodes. *Electrochim. Acta* **320**, 134561 (2019).

24. F. Malara, M. Fracchia, H. Kmentová, R. Psaro, A. Vertova, D. Oliveira de Souza, G. Aquilanti, L. Olivi, P. Ghigna, A. Minguzzi, A. Naldoni, Direct observation of photoinduced higher oxidation states at a semiconductor/electrocatalyst junction. *ACS Catal.* **10**, 10476–10487 (2020).

25. A. Tsyganok, P. Ghigna, A. Minguzzi, A. Naldoni, V. Murzin, W. Caliebe, A. Rothschild, D. S. Ellis, Operando X-ray absorption spectroscopy (XAS) observation of photoinduced oxidation in FeNi (oxy)hydroxide overlayers on hematite (α-Fe_2O_3) photoanodes for solar water splitting. *Langmuir* **36**, 11564–11572 (2020).

26. A. Minguzzi, A. Naldoni, O. Lugaresi, E. Achilli, F. D'Acapito, F. Malara, C. Locatelli, A. Vertova, S. Rondinini, P. Ghigna, Observation of charge transfer cascades in α-Fe_2O_3/IrO_x photoanodes by operando X-ray absorption spectroscopy. *Phys. Chem. Chem. Phys.* **19**, 5715–5720 (2017).

27. L. Li, J. Yang, H. Ali-Löytty, T. C. Weng, F. M. Toma, D. Sokaras, I. D. Sharp, A. Nilsson, Operando observation of chemical transformations of iridium oxide during photoelectrochemical water oxidation. *ACS Appl Energy Mater* **2**, 1371–1379 (2019).

28. L. Xi, C. Schwanke, D. Zhou, D. Drevon, R. Van De Krol, K. M. Lange, In situ XAS study of CoBi modified hematite photoanodes. *Dalton Trans.* **46**, 15719–15726 (2017).

29. A. Minguzzi, O. Lugaresi, C. Locatelli, S. Rondinini, F. D'Acapito, E. Achilli, P. Ghigna, Fixed energy X-ray absorption voltammetry. *Anal. Chem.* **85**, 7009–7013 (2013).

30. L. Liardet, J. E. Katz, J. Luo, M. Grätzel, X. Hu, An ultrathin cobalt-iron oxide catalyst for water oxidation on nanostructured hematite photoanodes. *J. Mater. Chem. A. Mater.* **7**, 6012–6020 (2019).

31. P. Vecchi, F. Ruani, M. Mazzanti, Q. R. Loague, R. Mazzaro, F. Boscherini, B. Ventura, G. J. Meyer, N. Armaroli, S. Caramori, L. Pasquini, Impact of Co-Fe overlayers on charge carrier dynamics at WO_3/$BiVO_4$ heterojunctions: A picosecond-to-second spectroscopic analysis. *ACS Energy Lett* **9**, 2193–2200 (2024).

32. P. Vecchi, A. Piccioni, R. Mazzaro, M. Mazzanti, V. Cristino, S. Caramori, L. Pasquini, Charge separation efficiency in WO_3/$BiVO_4$ photoanodes with CoFe prussian blue catalyst studied by wavelength-dependent intensity-modulated photocurrent spectroscopy. *Solar RRL* **6**, 2200108 (2022).

33. A. Piccioni, P. Vecchi, L. Vecchi, S. Grandi, S. Caramori, R. Mazzaro, L. Pasquini, Distribution of relaxation times based on lasso regression: A tool for high-resolution analysis of IMPS data in photoelectrochemical systems. *J. Phys. Chem. C* **127**, 7957–7964 (2023).

34. C. Zachäus, F. F. Abdi, L. M. Peter, R. Van De Krol, Photocurrent of $BiVO_4$ is limited by surface recombination, not surface catalysis. *Chem. Sci.* **8**, 3712–3719 (2017).

35. F. T. Haase, A. Bergmann, T. E. Jones, J. Timoshenko, A. Herzog, H. S. Jeon, C. Rettenmaier, B. R. Cuenya, Size effects and active state formation of cobalt oxide nanoparticles during the oxygen evolution reaction. *Nat. Energy* **7**, 765–773 (2022).

36. H. Dau, P. Liebisch, M. Haumann, X-ray absorption spectroscopy to analyze nuclear geometry and electronic structure of biological metal centers-potential and questions examined with special focus on the tetra-nuclear manganese complex of oxygenic photosynthesis. *Anal. Bioanal. Chem.* **376**, 562–583 (2003).

37. D. A. Kuznetsov, B. Han, Y. Yu, R. R. Rao, J. Hwang, Y. Román-Leshkov, Y. Shao-Horn, Tuning redox transitions via inductive effect in metal oxides and complexes, and implications in oxygen electrocatalysis. *Cell Press* **2**, 225–244 (2018).

38. K. Li, D. Xue, Estimation of electronegativity values of elements in different valence states. *J. Phys. Chem. A* **110**, 11332–11337 (2006).

39. Q. Shi, S. Murcia-López, P. Tang, C. Flox, J. R. Morante, Z. Bian, H. Wang, T. Andreu, Role of tungsten doping on the surface states in $BiVO_4$ photoanodes for water oxidation: Tuning the electron trapping process. *ACS Catal.* **8**, 3331–3342 (2018).

40. L. Bertoluzzi, L. Badia-Bou, F. Fabregat-Santiago, S. Gimenez, J. Bisquert, Interpretation of cyclic voltammetry measurements of thin semiconductor films for solar fuel applications. *J. Phys. Chem. Lett.* **4**, 1334–1339 (2013).

41. A. M. Ullman, C. N. Brodsky, N. Li, S. L. Zheng, D. G. Nocera, Probing edge site reactivity of oxidic cobalt water oxidation catalysts. *J. Am. Chem. Soc.* **138**, 4229–4236 (2016).

42. H. Y. Wang, S. F. Hung, Y. Y. Hsu, L. Zhang, J. Miao, T. S. Chan, Q. Xiong, B. Liu, In situ spectroscopic identification of μ-OO bridging on spinel Co_3O_4 water oxidation electrocatalyst. *J. Phys. Chem. Lett.* **7**, 4847–4853 (2016).

43. M. Zhang, M. de Respinis, H. Frei, Time-resolved observations of water oxidation intermediates on a cobalt oxide nanoparticle catalyst. *Nat. Chem.* **6**, 362–367 (2014).

44. A. J. Kaufman, A. C. Nielander, G. J. Meyer, S. Maldonado, S. Ardo, S. W. Boettcher, Absolute band-edge energies are over-emphasized in the design of photoelectrochemical materials. *Nat. Catal.* **7**, 615–623 (2024).

45. V. Cristino, S. Marinello, A. Molinari, S. Caramori, S. Carli, R. Boaretto, R. Argazzi, L. Meda, C. A. Bignozzi, Some aspects of the charge transfer dynamics in nanostructured WO_3 films. *J. Mater. Chem. A. Mater.* **4**, 2995–3006 (2016).

46. L. Meda, G. Tozzola, A. Tacca, G. Marra, S. Caramori, V. Cristino, C. Alberto Bignozzi, Photo-electrochemical properties of nanostructured WO_3 prepared with different organic dispersing agents. *Sol. Energ. Mat. Sol. C.* **94**, 788–796 (2010).

47. V. Cristino, L. Pasti, N. Marchetti, S. Berardi, C. A. Bignozzi, A. Molinari, F. Passabi, S. Caramori, L. Amidani, M. Orlandi, N. Bazzanella, A. Piccioni, J. Kopula Kesavan, F. Boscherini, L. Pasquini, Photoelectrocatalytic degradation of emerging contaminants at WO_3/$BiVO_4$ photoanodes in aqueous solution. *Photochem. Photobiol. Sci.* **18**, 2150–2163 (2019).

48. J. A. Seabold, K.-S. Choi, Efficient and stable photo-oxidation of water by a bismuth vanadate photoanode coupled with an iron oxyhydroxide oxygen evolution catalyst. *J. Am. Chem. Soc.* **134**, 2186–2192 (2012).

49. F. d'Acapito, G. O. Lepore, A. Puri, A. Laloni, F. La Manna, E. Dettona, A. De Luisa, A. Martin, The LISA beamline at ESRF. *J. Synchrotron Radiat.* **26**, 551–558 (2019).

50. R. Memming, *Semiconductor Electrochemistry* (Wiley, 2000).

51. B. Hirschorn, M. E. Orazem, B. Tribollet, V. Vivier, I. Frateur, M. Musiani, Determination of effective capacitance and film thickness from constant-phase-element parameters. *Electrochim. Acta* **55**, 6218–6227 (2010).

52. J. Timoshenko, B. Roldan Cuenya, In situ/operandoelectrocatalyst characterization by X-ray absorption spectroscopy. *Chem. Rev.* **121**, 882–961 (2021).
